# Medicinal and Aromatic Plant Oils in Aquafeeds: Mechanistic Perspectives on Growth Promotion, Immunomodulation, and Stress Resilience

**DOI:** 10.1155/anu/8992384

**Published:** 2026-05-05

**Authors:** Mustafa Öz, Enes Üstüner, Sümmani Çifci

**Affiliations:** ^1^ Department of Fisheries and Diseases, Faculty of Veterinary Medicine, Aksaray University, Aksaray, Türkiye, aksaray.edu.tr; ^2^ Department of Veterinary Parasitology, Faculty of Veterinary Medicine, Aksaray University, Aksaray, Türkiye, aksaray.edu.tr

**Keywords:** aquaculture, growth performance, immune system modulation, medicinal and aromatic plant oils (MAPOs), phytobiotics, sustainable aquafeeds

## Abstract

The aquaculture industry is increasingly transitioning toward sustainable aquafeeds, driven by the economic and environmental necessity to replace marine‐derived fishmeal and fish oil with plant‐ and insect‐based alternatives. This nutritional shift introduces physiological challenges, accelerating the search for natural, sustainable functional additives. The primary goal of this review is to comprehensively evaluate the application of medicinal and aromatic plant oils (MAPOs) in aquafeeds, providing mechanistic perspectives on their roles in growth promotion, immunomodulation, and stress resilience. We synthesize current literature to link MAPO chemical composition, particularly phenolic monoterpenes and phenylpropenes, with biological responses, advanced delivery systems, and metabolic pathways. Key findings demonstrate that MAPOs can effectively stimulate appetite, modulate the gut microbiome, and enhance antioxidant defenses via the Nrf2‐Keap1 pathway, thereby improving disease resistance. However, a critical limitation in the current literature is the high variability and inconsistent outcomes reported across different aquatic species and developmental stages. These discrepancies are largely attributed to strong chemotypic variability of essential oils, unstandardized extraction protocols, and dose‐dependent responses that can sometimes yield neutral or suppressive effects. To successfully transition MAPOs from experimental trials to reliable commercial applications, future research must prioritize standardized dose–response evaluations, address species‐specific variability, and utilize advanced formulation technologies such as nanoemulsions.

## 1. Introduction

Aquaculture nutrition is undergoing a structural transformation driven by a declining reliance on marine‐derived fishmeal (FM) and fish oil (FO). Volatility in FM/FO pricing, ecological concerns, and sustainability constraints have accelerated the diversification of aquafeeds. Consequently, the industry is shifting toward alternative ingredients such as plant‐based proteins, insect meals, microalgae, and agro‐industrial by‐products [1–3]. To counter the physiological and metabolic challenges posed by these alternative diets, the application of targeted functional feed additives has become essential. For instance, recent advancements demonstrate that supplementing specific functional nutrients, such as glutamine, can significantly improve growth performance, fortify antioxidant defenses, and enhance the ultimate sensory quality of fillets in commercially valuable species like rainbow trout [4]. In parallel with these targeted single‐nutrient interventions, complex botanical derivatives and MAPOs are increasingly being explored for their broad‐spectrum biological benefits. Although these substitutions reduce dependence on marine resources, they introduce new physiological challenges to farmed fish, including amino acid imbalances, antinutritional factors, altered gut integrity, chitin digestibility, and reduced long‐chain n‐3 polyunsaturated fatty acids (PUFAs) [5–7]. These nutritional tradeoffs translate into growth reduction, compromised immunity, and poorer feed utilization, issues that cannot be fully mitigated without targeted nutritional strategies. Emerging technologies such as protein fermentation, lipid fortification, enzymatic processing, and functional additives attempt to address these constraints, yet their cost and scalability remain limiting in commercial aquaculture [1, 7].

Beyond economic pressures, regulatory and consumer expectations reshape feed development. Antibiotic growth promoters are increasingly restricted or banned across many markets, most notably under EU Regulation 1831/2003, due to food safety concerns and antimicrobial resistance (AMR) [8]. Coupled with rising demand for antibiotic‐free, eco‐friendly aquaculture products, these regulatory shifts have accelerated the adoption of natural additives such as probiotics, phytogenics, prebiotics, yeast, and carotenoid‐based functional ingredients [9–11]. Although current advances in probiotics, synbiotics, microalgae, and fermentation‐based products have improved gut health and feed conversion, their effectiveness remains species‐ and dosage‐dependent, and they do not consistently provide robust immune protection comparable to antibiotics in stressful production environments [12–14].

In recent years, an expanding body of literature highlights the profound impact of medicinal plants and their diverse derivatives such as polyphenols, fruit and seed extracts, and natural pigments on enhancing growth performance, physiological homeostasis, and immunity in aquatic animals. Dietary polyphenols and polyphenol‐rich additives, in particular, are increasingly recognized for their robust antioxidant and health‐promoting benefits in aquaculture [15]. Specific derivations, such as grape seed and cornelian cherry (*Cornus mas* L.) fruit extracts, have been shown to significantly improve growth metrics, humoral and mucosal immunity, and resistance against *Aeromonas hydrophila* in common carp (*Cyprinus carpio*) [16, 17]. Furthermore, *Plantago ovata* seed extracts have proven effective in mitigating the immunosuppressive effects of severe environmental stressors, such as ammonia toxicity, while preserving immune function in carp fingerlings [18]. Beyond seed extracts, functional fruits and rhizomes like amla (*Phyllanthus emblica*) and ginger (*Zingiber officinale*) positively alter biochemical parameters, fortify skin mucosal and serum immunities, and upregulate antioxidant and growth‐related gene expressions in species like Nile tilapia and zebrafish [19, 20]. Additionally, functional plant‐derived pigments play a crucial role in regulating blood‐digestive physiology and modulating inflammatory gene transcription, thereby enhancing resistance to bacterial infections such as *Pseudomonas aeruginosa* [21]. These broad‐spectrum benefits of plant derivatives lay a strong foundation for understanding more concentrated phytogenic applications.

Within this broader trend, medicinal and aromatic plant oils (MAPOs) have attracted particular scientific interest. These oils comprise phenolic monoterpenes (e.g., thymol and carvacrol), phenylpropenes (e.g., eugenol and cinnamaldehyde), and monoterpene alcohol/aldehyde compounds (e.g., linalool and citral), which exhibit broad biological activities relevant to aquaculture. Controlled supplementation of essential oils derived from oregano, thyme, clove, or cinnamon has demonstrated improvements in growth performance, digestive enzyme activity, feed conversion ratio (FCR), innate immune responses, and survival following pathogenic challenge [22, 23]). These findings collectively position MAPOs not simply as feed flavoring agents but as mechanistically active ingredients that can modulate inflammatory pathways, antioxidant systems, endocrine control, and microbiome composition.

Despite the notable progress, however, fundamental limitations hinder scientific consistency and commercial translation. Essential oils exhibit strong chemotypic variability, influenced by species genotype, geographic origin, irrigation, harvesting season, and extraction methods [24–26]. Hydrodistillation yields unstable volatile fractions, whereas supercritical CO_2_ extraction produces thermally stable extracts with improved delivery potential [26–28]. The absence of standardized chromatographic fingerprinting, Good Agricultural and Collection Practices (GACP), and Good Manufacturing Practices (GMP) complicates reproducibility and hinders dose–response optimization [25, 29]. Consequently, many studies test short experimental windows, small sample sizes, or inconsistent immunological endpoints, leading to inconclusive dose thresholds and noncomparable outcomes.

At the biological level, MAPOs interact with fish physiology through multilayered mechanisms. Phenolic compounds act as radical scavengers and metal chelators in aquafeeds, inhibiting lipid peroxidation in ways comparable to synthetic antioxidants such as BHT/BHA [30–32]. Their bactericidal action against pathogens such as *Vibrio*, *Aeromonas*, and *Streptococcus* is linked to membrane disruption, permeability changes, and oxidative destabilization [31, 33]. The synergy between essential oils further enhances antimicrobial and antioxidant effects, yet the underlying rules remain poorly mapped and species‐/matrix‐specific [30, 34]. In parallel, the lipophilic nature of these oils confers advantages for intestinal absorption and lymphatic transport, particularly when delivered via nanoemulsions, self‐emulsifying drug delivery systems (SEDDS), liposomal carriers, or lipid–drug conjugates [2, 35–39].

Given the combined scientific, operational, and regulatory pressures on aquafeed innovation, MAPOs represent a promising class of functional technologies. However, current literature lacks a unifying framework that integrates (i) biochemical composition and chemotype variability, (ii) delivery system engineering, (iii) molecular growth and immune pathways, and (iv) real‐world commercial constraints. Most reviews treat these domains separately, overlooking interactions between extraction methods, feed processing, microbial regulation, endocrine signaling, antioxidant defense, and disease resilience. Addressing this gap is essential to transform MAPO research from case‐specific supplementation trials into mechanistically guided, industry‐ready formulations.

Accordingly, the present review synthesizes MAPO functions through a mechanistic continuum, linking oil chemistry and extraction technologies to digestive enzymes, gut microbiota, oxidative stress pathways, neuroendocrine regulation, and mucosal/systemic immunity in farmed aquatic species. We further evaluate MAPO performance in sustainable aquafeed systems, regulatory environments, and practical delivery strategies, while identifying research priorities to standardize chemotype documentation, optimize dosing, and accelerate industrial translation.

### 1.1. Literature Search Strategy and Selection Criteria

To comprehensively synthesize the current state of knowledge regarding MAPO applications in aquafeeds, a systematic literature search was conducted utilizing major academic databases, including Web of Science, Scopus, PubMed, and Google Scholar. The search encompassed peer‐reviewed articles published primarily between 2010 and 2025 to capture the most recent advancements in the field. The primary search queries included combinations of keywords such as “medicinal and aromatic plant oils,” “essential oils,” “phytobiotics,” “aquaculture,” “aquafeed,” “growth performance,” “immunomodulation,” and “stress resilience.” Inclusion criteria were limited to English‐language, peer‐reviewed original research articles and comprehensive reviews that evaluated the in vivo dietary application of MAPOs or provided in vitro mechanistic insights relevant to aquatic species. Studies lacking clear descriptions of oil extraction methods, undefined bioactive compound profiles, or those focusing exclusively on terrestrial livestock without comparative aquaculture relevance were excluded to maintain the specific focus and rigor of this review.

## 2. Chemical and Functional Properties of Medicinal and Aromatic Plant Oils

### 2.1. Major Bioactive Compounds

**Figure 1:**
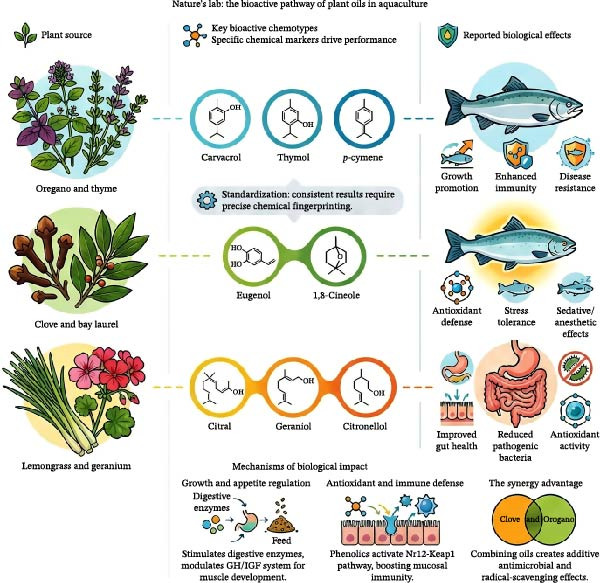
Conceptual framework linking major medicinal and aromatic plant sources (e.g., oregano, cinnamon, clove, and thyme) to their primary bioactive chemotypes, target physiological systems, and functional biological outcomes in aquaculture.

In aquaculture, a wide range of medicinal and aromatic plant oils have been used, and numerous physiological, pathological, and biochemical effects associated with their application have been reported [40–44]. The levels of secondary metabolites in plant oils depend on three main factors, which include seasonal patterns and plant genotypes and harvesting techniques. The control of seasonal elements and irrigation systems and harvest schedules becomes essential because terpenoid and phenolic marker levels follow particular patterns that depend on both time and location [24–26]. The extraction and formulation techniques applied to these oils result in changes to their chemical structure and biological characteristics. The hydrodistillation process generates volatile compounds with different chemical profiles, but SC‐CO_2_ extraction produces stable extracts that maintain their chemical structure when exposed to heat, and these extracts become more suitable for aquatic systems through microencapsulation and nanoemulsification processes [26–28]. Effective standardization requires rigorous chemotype and harvest control systems. This involves implementing GACP alongside GMP. Furthermore, consistent bioactive loads must be ensured through chromatographic fingerprinting (GC–MS or HPLC) and strict batch release criteria [24, 25, 29]. The scientific community has extensively researched carvacrol and thymol (phenolic monoterpenes) because they demonstrate strong antimicrobial and antioxidant and immunostimulant properties. The phenylpropanoid compound eugenol shows antimicrobial activity while also functioning as an anesthetic substance. The effectiveness of these compounds depends on species type and dosage amount and formulation method, which requires individual validation for each target species [45–48]. The development of commercially viable methods requires SC‐CO_2_ extraction to work with distillation techniques and affordable formulation methods that include microencapsulation and nanoemulsions and quality control systems for batch certification [26, 28, 49, 50]. The major medicinal and aromatic plant oils used in aquafeeds, along with their principal bioactive compounds and reported biological effects, are summarized in Table 1. To further clarify chemotypic variability and its biological significance, a schematic representation linking major plant sources to their primary bioactive compounds, target physiological systems, and subsequent biological outcomes is provided in Figure 1.

**Table 1:** Major medicinal and aromatic plant oils used in aquafeeds and their principal bioactive compounds and biological effects.

Plant species (common name, scientific name)	Main bioactive compounds	Reported biological effects in fish	Citations
Oregano (*Origanum vulgare*, *O. onites*)	Carvacrol, thymol, p‐cymene, γ‐terpinene	Growth promotion, improved feed efficiency, enhanced immunity (lysozyme, phagocytosis), antioxidant activity, disease resistance (*Aeromonas*, *Lactococcus*)	[51–59]
Thyme (*Thymus vulgaris*)	Thymol, carvacrol, p‐cymene, γ‐terpinene	Antioxidant, immunostimulant, improved growth, protection against malathion and *Aeromonas*, enhanced digestive enzymes	[53, 55, 60–63]
Savory (*Satureja hortensis*)	Carvacrol, thymol, γ‐terpinene	Growth, digestive enzyme activity, humoral immunity, resistance to *Aeromonas hydrophila*	[57, 60, 61]
Dill (*Anethum graveolens*)	Carvone, limonene, dillapiole	Antioxidant, improved immunity, growth promotion	[61, 64]
Mooseer (*Allium hirtifolium*)	Organosulfur compounds	Enhanced immunity, reduced mortality after bacterial challenge	[61]
Lemongrass (*Cymbopogon citratus*)	Citral (α‐citral, β‐citral), limonene	Growth, antioxidant, immune response, reduced pathogenic bacteria, improved gut health	[65, 66]
Geranium (*Pelargonium graveolens*)	Citronellol, geraniol	Growth, antioxidant, immune response, reduced pathogenic bacteria	[65]
Bay Laurel (*Laurus nobilis*)	1,8‐cineole, linalool, eugenol	Growth, immunity, antioxidant defense, improved stress tolerance	[59, 64]
Clove (*Syzygium aromaticum*)	Eugenol	Sedative/anesthetic, antioxidant, antibacterial, improved growth	[53, 67, 68]
Peppermint (*Mentha piperita*)	Menthol, menthone, menthyl acetate	Antioxidant, immune modulation, improved gut health, stress reduction	[66, 68–70]
Tea Tree (*Melaleuca alternifolia*)	Terpinen‐4‐ol, γ‐terpinene, α‐terpinene	Antimicrobial, complement activation, improved gut morphology	[63, 66, 70, 71]
Curcuma (*Curcuma aromatica*, *C. longa*)	Curcuminoids, turmerones	Growth, immunity, resistance to *Aeromonas*, anti‐inflammatory	[72, 73]
Myrrh (*Commiphora myrrha*)	Furanoeudesma‐1,3‐diene, curzerene	Immunomodulation, increased survival after *Aeromonas* challenge	[74]
Black cumin (*Bunium persicum*)	Cuminaldehyde, p‐cymene, limonene, γ‐terpinene	Enhanced immunity, reduced mortality after *Aeromonas* challenge	[75]
Juniper (*Juniperus communis*)	α‐pinene, sabinene, myrcene	Growth, digestive enzymes, antioxidant, immune enhancement	[76]
Lippia (*Lippia alba*, *L. origanoides*, *L. sidoides*)	Geranial, limonene, neral, thymol, carvacrol	Antioxidant, antibacterial (*Vibrio*), sedative, stress reduction	[66, 77]
Citrus (*C. limon*, *C. sinensis*)	Limonene, citral, linalool	Growth, immunity, disease resistance (*Edwardsiella*, *Streptococcus*), antioxidant	[78–81]
Rosemary (*Rosmarinus officinalis*)	1,8‐cineole, camphor, *α*‐pinene	Antioxidant, antibacterial, improved growth	[53, 64, 67]
Pine (*Pinus thunbergii*, *Abies holophylla*)	α‐pinene, bornyl acetate, camphene	Antibacterial (*Edwardsiella*, *Photobacterium*), potential antibiotic alternative	[82]
Blend/commercial mixes (e.g., carvacrol, thymol, limonene, cineole, pinene)	Multiple	Synergistic effects: growth, immunity, antioxidant, gut health	[56, 83, 84]

### 2.2. Antioxidant and Antimicrobial Mechanisms

Plant oils containing phenolic monoterpene and polyphenol compounds such as oregano and thyme and clove oils show similar lipid oxidation inhibition in aquafeed through their ability to scavenge radicals and chelate metals and break down fatty acid chains similar to BHT/BHA. Research studies conducted through in vitro methods and storage tests demonstrate these oils function similarly to BHT/BHA, but their performance depends on the amount used and the type of feed and methods of preparation [30–32]. Thymol/carvacrol‐rich and eugenol‐rich oils show broad bactericidal activity and have been reported to reduce *Vibrio*, *Aeromonas*, and *Streptococcus* burdens or disease outcomes in aquaculture studies and reviews [31, 33]. Essential oils need special dosing methods and encapsulation techniques because their lipophilic/volatile nature and membrane‐perturbing mechanism require specific methods to prevent gut exposure, which leads to microbiota modulation instead of long‐term depletion when optimized correctly [31, 50]. The combination of oils produces stronger radical‐scavenging and Fe‐chelating and anti‐lipid‐peroxidation effects because of both additive and synergistic mechanisms (e.g., clove + coriander, ginger + hop). The synergistic effects of oil combinations depend on specific oil compositions and dosages and matrix types, which need separate validation through orthogonal assays [30, 34].

### 2.3. Lipid‐Solubility Advantages

**Figure 2:**
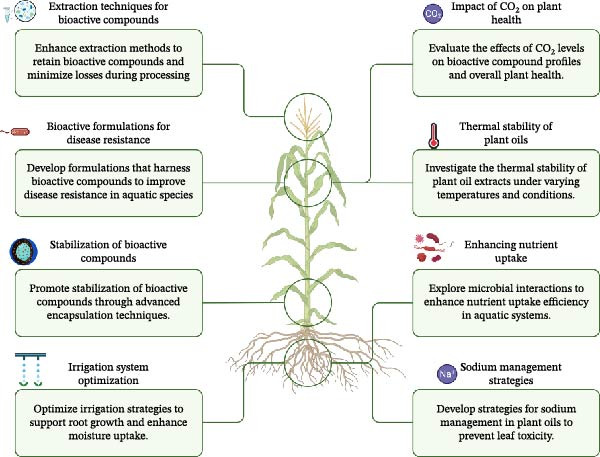
Schematic representation of extraction techniques and formulations for enhancing bioactive compounds in medicinal and aromatic plant oils. This figure illustrates various methods such as hydrodistillation and supercritical CO_2_ extraction and their effects on the retention and bioavailability of key phytochemicals like carvacrol, thymol, and eugenol. It also highlights the applications of these compounds in improving disease resistance, stabilizing bioactive properties, enhancing nutrient uptake efficiency, and managing sodium levels to prevent toxicity in aquatic systems. The figure serves as a comprehensive overview to understand how these extraction methods impact the functional properties of plant oils in aquaculture and beyond.

Plant oils can pass through intestinal lipid/micellar phases because of their lipophilic nature, which allows them to move freely through cells and potentially reach the lymphatic system. The oral bioavailability of water‐soluble extracts becomes less effective because they need to diffuse through water, whereas lipid‐based formulations and nanocarriers enhance absorption rates. The absorption of unformulated terpenes remains low because they fail to distribute correctly, and they cannot receive encapsulation [35–37, 85, 86]. The optimal carriers for fish feed delivery include nanoemulsions and SEDDS and liposomes and SLN/NLC and oleogels and lipid–drug conjugates. The choice of carrier needs to be determined through species‐specific in vitro digestion tests and Caco‑2 cell assays and residue measurement studies [39, 87, 88]. The amount of inclusion affects the availability of fatty acids and lipophilic phytochemicals, which changes how tissues use fatty acids and affects membrane properties through dose‐specific and species‐related mechanisms. The study of essential oil effects on trout lipid use needs lipidomic analysis and membrane‐fluidity measurement to understand the process [39, 47]. The amount of tissue accumulation depends on both the formulation type and the species being studied: plant oils show minimal fish tissue retention, but lipid carriers and conjugation methods improve their ability to reach systemic circulation and the lymphatic system and organs which requires experimental tissue‐residue and functional testing [29, 36, 45, 50]. Molecular insights into the efficacy of medicinal and aromatic plant oils are summarized in Figure 2, which highlights the extraction techniques and their impact on bioactive compounds.

## 3. Mechanisms of Growth Enhancement

### 3.1. Appetite Stimulation Pathways

Phytobiotics, obtained from the extracts and oils (essential oils) of medicinal plants, have appetite‐enhancing properties in aquatic organisms [89]. These properties contribute to the acceleration of growth and the development of the immune system in aquatic organisms. Plant essential oils are prominent in appetite regulation [90, 91]. In particular, the addition of essential oils to the diet enhances palatability and regulates appetite control genes in the hypothalamus‐pituitary gland [45, 89]. The positive effects of dietary vegetable oils on growth are closely similar to those of prebiotics. This similarity is primarily associated with their positive effects on the gut microbiota [10].

### 3.2. Digestive Enzyme Secretion and Gut Microbiome Modulation

The health of the gastrointestinal microbiota is a crucial element that can directly impact the overall health of the host. The gut microbiota is a complex network of microorganisms that is part of the fish’s intestinal tract and directly responsible for food processing [92]. Therefore, interventions that can affect intestinal integrity and microbiota composition are crucial for the living organism.

The structure of the microbiota is closely related to the diet [93]. Aromatic and medicinal plant oils added to the diet affect the microbiota in various ways [94]. Depending on the affected microbial activity, various specific metabolites are produced. These metabolites can have a direct impact on digestive processes. As a result of this effect, changes occur in the secretory functions of enterocytes. The secretion of enzymes, especially intestinal peptides, is triggered [95]. Due to this stimulation, an increase in intestinal motility and enzyme activity occurs, which in turn affects growth by increasing the rate and amount of nutrient absorption.

### 3.3. Fish Metabolism and Endocrine Regulation

The gut microbiota secretes metabolites in response to substrates present in the intestinal lumen [95]. These metabolites can regulate the intestinal barrier, absorption mechanisms, and nutrient uptake and storage processes via enterocytes [96]. This regulation directly affects both carbohydrate and fat metabolism. Furthermore, vegetable oils contribute to protein metabolism through their positive effects on digestion and absorption, increasing the amino acid synthesis sequence required for protein synthesis and contributing to fish growth through muscle development [10]. Moreover, the dietary application of specific functional botanical extracts has proven highly effective in optimizing somatic growth and physiological health. For example, recent trials have demonstrated that supplementing Nile tilapia diets with artichoke (*Cynara scolymus*) leaf extract significantly improves the FCR and specific growth rate (SGR), while concurrently enhancing hematological indices and blood biochemistry [40]. Beyond somatic growth and physiological health, such functional dietary interventions are also crucial for optimizing the final product quality. For instance, targeted amino acid supplementation, such as glutamine, has been proven to significantly elevate the beneficial n‐3/n‐6 PUFA ratio, improve meat color indices, and enhance the overall fillet lipid profile in species like rainbow trout, aligning with modern consumer demands for high‐quality aquaculture products [97].

As in vertebrates, growth in fish is primarily mediated by growth hormone (GH) and insulin‐like growth factor (IGF). This system, called the GH/IGF system in fish, simply produces its effect by regulating the synthesis of IGF‐1 in tissues and organs such as liver, muscle, and gills by circulating GH. Upon stimulation of IGF‐1 synthesis, IGF, secreted through IGF receptors (IGFR), acts on target tissues, promoting growth and differentiation in target tissues and cells [98].

**Figure 3:**
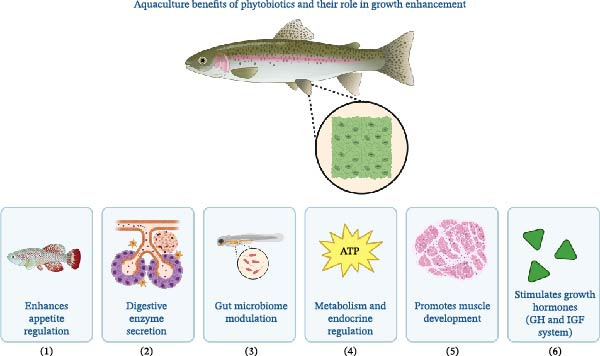
Mechanisms of growth enhancement through phytobiotics in aquaculture. This figure illustrates the pathways, including: (1) appetite regulation via appetite‐enhancing properties, (2) secretion of digestive enzymes, (3) modulation of gut microbiome, (4) metabolism and endocrine regulation affecting nutrient uptake, (5) promotion of muscle development, and (6) stimulation of growth hormones (GH and IGF system). These mechanisms collectively contribute to improved growth performance in aquatic organisms.

The potential effects of plant‐based oils on the GH axis have been suggested by several studies, although many of the precise molecular interactions remain hypothetical rather than experimentally validated. Research in this field has shown that the expression levels of growth factor genes (IGF‐1 and IGF‐2) at the mRNA level may support the positive effects of plant products on growth regulation, yet responses can vary significantly depending on the fish species and oil composition [23, 45, 99–101]. The role of phytobiotics in enhancing growth mechanisms in aquaculture is illustrated in Figure 3.

## 4. Immunomodulatory Mechanisms

### 4.1. Innate Immune System Modulation

Plant oils containing essential oils and lipid extracts boost lysozyme and complement activity through three main pathways, which include direct activation of innate immunity and stress protection that stops complement system dysfunction and microbial gut regulation for enhanced systemic defense readiness. Research shows that lemongrass essential oil (LEO) and geranium essential oil (GEO) increase Nile tilapia plasma lysozyme levels, according to Al‐Sagheer et al. [65]. The same effect on innate immune response has been achieved through herbal supplements in rainbow trout, according to Fadeifard et al. [102]. The research indicates essential oils help reduce stress while protecting complement proteins, which become damaged when fish experience stress during aquaculture, according to Souza et al. [103]. The gut‐immune connection provides an alternative method to boost immune function according to Kelly and Salinas [104]. Research about myeloperoxidase (MPO) today does not provide direct evidence, although studies indicate terpenes such as citral and geraniol and gingerols and thymoquinone and limonene and 1,8‐cineole protect innate immune markers and reduce stress in dietary studies and sedative research [65, 102, 103, 105, 106]. Plant oils have been shown to boost macrophage phagocytic activity and respiratory burst through their ability to produce lysozyme opsonins and antioxidants while activating the microbiome, but the effects depend on the specific plant species and the amount used. The study found that particular doses of LEO (200 and 400 mg) and GEO (400 mg) resulted in elevated lysozyme production [65, 102–104]. The results need verification through production‐level dose–response validation tests to establish their accuracy [73].

### 4.2. Adaptive Immunity Support

The head kidney and spleen operate as main lymphoid organs according to the corpus, but teleosts and cartilaginous fishes develop germinal‐center‐like structures that enable affinity maturation and memory development [107–110]. Research shows that adjuvants and antigens and dietary immunomodulators affect vaccine responses through changes in MHC expression and CD^4^/CD^8^ ratios and cytokine production and spleen/head‐kidney leukocyte distribution (examples: rtOmpF + ISA763, live/attenuated strains, dietary tryptophan) [111–115]. The provided references do not specify plant‐oil bioactives that have proven lymphocyte‐proliferative or T‐cell‐activating effects so no plant‐oil dosing/timing recommendations can be made based on these findings [111, 114, 115]. The operational phase of aquaculture trials requires controlled dose–response studies to evaluate adjuvant and booster effects, and organ‐specific measurements (spleen/head‐kidney gene expression and leukocyte counts and antibody titers) before moving to field implementation [111, 112, 116].

### 4.3. Mucosal Immunity

**Figure 4:**
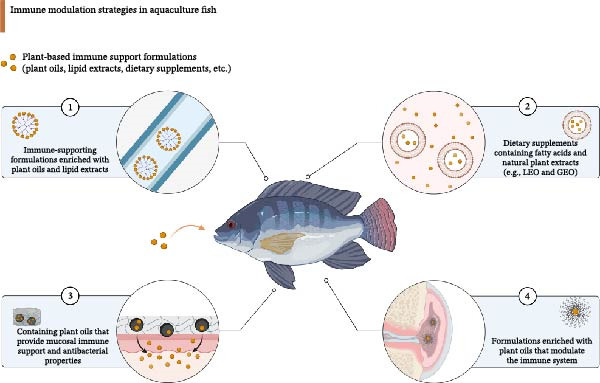
Immune modulation strategies in aquaculture fish, illustrating various approaches to enhance immune responses. The strategies include: (1) development of immune‐supporting formulations enriched with plant oils and lipid extracts, (2) incorporation of dietary supplements containing fatty acids and natural plant extracts (e.g., LEO and GEO), (3) utilization of plant oils that provide mucosal immune support and antibacterial properties, and (4) formulations enriched with plant oils that modulate the immune system. These approaches aim to improve the overall health and disease resistance of aquaculture fish.

• Research indicates that plant essential oils added to food increase mucus and AMP production in skin and intestinal tissues, which produces mucus that contains lysozyme and MPO and proteases and immunoglobulins and antimicrobial peptides through modified secretory pathways and increased mucin/AMP gene expression [75, 117, 118].
• The intestinal barrier and translocation process produce species‐dependent results that show that *Bunium persicum* essential oils combined with Artemisia (tarragon) and oregano extracts containing phenols carvacrol and thymol reduce enteric infections and mortality rates by strengthening mucus barriers and reducing pathogen spread [75, 118].
• Research on direct oil application to gills remains scarce because gills function as mucosal immune organs, which makes it probable that oil benefits for skin and gut mucus also protect gill mucus and AMPs and respiratory health, thus requiring specific studies on gill mucus and AMP and histology responses to different doses [119, 120].
• The skin barrier and repair mechanisms need essential oils to trigger mucin production and activate antimicrobial enzymes and proteases and alter secretory pathways, which decrease microbial numbers and fix the barrier function. The commercial production process needs species‐specific dose–response feeding studies that monitor mucosal biomarkers to confirm treatment effectiveness before beginning large‐scale manufacturing [75, 117, 120]. The immune modulation strategies in aquaculture fish, utilizing plant‐based formulations and supplements, are depicted in Figure 4.

## 5. Antioxidant Mechanisms

### 5.1. ROS Scavenging and Nrf2‐Keap1 Pathway

**Figure 5:**
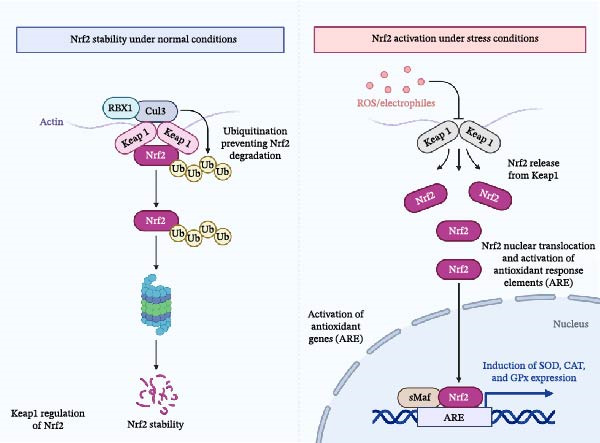
The Nrf2 signaling pathway in aquaculture fish demonstrates its regulation under constitutive conditions and oxidative stress. Under normal conditions, Keap1 mediates the degradation of Nrf2, maintaining low levels of this transcription factor. In contrast, under oxidative stress, ROS and electrophilic compounds disrupt the Keap1–Nrf2 interaction, leading to Nrf2 release, nuclear translocation, and activation of antioxidant response elements (ARE). This process ultimately enhances the expression of antioxidant enzymes such as superoxide dismutase (SOD), catalase (CAT), and glutathione peroxidase (GPx), aiding in cellular protection against oxidative damage.

Plant‐oil phenolics activate the Keap1–Nrf2 axis in farmed fish by transforming into electrophilic compounds. These compounds prevent Keap1 from tagging cysteine residues for ubiquitination, thereby maintaining Nrf2 stability. Once Nrf2 enters the nucleus, it triggers the activation of ARE‐dependent antioxidant enzymes, including SOD, CAT, and GPx. Recent toxicological models strongly support this mechanism; for instance, dietary supplementation with black cumin (*Nigella sativa*) oil has been shown to effectively restore SOD, CAT, and GPx activities and alleviate severe oxidative damage in Nile tilapia exposed to chemical stressors, such as waterborne boric acid toxicity [121]. The electrophile–Keap1 mechanism and Nrf2 regulation pathway exist for dietary polyphenols and operate similarly in teleost fish species [122, 123]. The research on aquaculture shows that phenolic‐rich oils/extracts containing tea polyphenols and grape‐seed and rosemary and phenolic essential oils effectively neutralize free radicals while activating Nrf2 pathways. The study on crowded carp shows that tea polyphenols activate Nrf2/Keap1 signaling pathways while restoring T‐AOC and SOD and CAT and GSH enzyme activities [124, 125]. The oils operate through two separate mechanisms that remove ROS and activate Nrf2 to produce GPx enzymes that reduce MDA levels and prevent lipid peroxidation and ferroptosis from happening in fish tissues under high‐intensity farming systems [126]. The activation process happens through Keap1 cysteine modification and p62‐mediated Keap1 sequestration and disruption of Keap1–Nrf2 binding, which results in PPARα/NF‐κB cross‐talk that modifies lipid metabolism and inflammation to boost cellular resistance [127]. The activation of the Nrf2 signaling pathway in aquaculture fish through dietary polyphenols is illustrated in Figure 5.

### 5.2. Upregulation of Antioxidant Enzymes

Plant‑oil phenolics activate SOD and CAT and GPx expression through electrophilic (bio)conversion, which results in Keap1 cysteine modification to stabilize Nrf2 and initiate ARE‑dependent transcription of antioxidant enzymes. The Keap1–Nrf2 mechanism exists in teleosts according to studies about fish consumption of polyphenols [65, 128, 129]. The empirical aquaculture data demonstrates that tea polyphenols and grape‐seed/rosemary extracts and essential oils (oregano, lemongrass, and geranium) exhibit powerful radical‐scavenging abilities and enzyme‐upregulating properties, but researchers have not conducted enough studies to establish their relative effectiveness [129–131]. The protective effects against lipid peroxidation result from two mechanisms of phenolic hydroxyls, which either directly donate hydrogen atoms to ROS or neutralize ROS while Nrf2 activation leads to GPx (including GPx4) and phase II enzyme production, which decreases hydroperoxide and MDA and ferroptotic damage in fish tissues [65, 132]. The responses depend on species type and dosage levels and developmental stages of the organisms: SOD/CAT/GPx enzyme levels increase in liver and intestine and gill and muscle tissues during short‐term juvenile tests with sea bass and tilapia and carp. The results show different patterns between adult and longer‐term studies and specific tissue types, so researchers need to test different species and ages and target specific tissues [128, 129, 133].

### 5.3. Mitochondrial Protection

**Figure 6:**
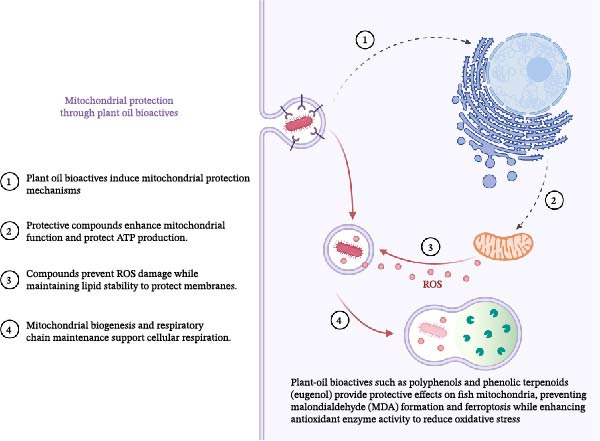
Mechanisms of mitochondrial protection through plant‐oil bioactives in aquaculture fish. The diagram highlights how these bioactive compounds induce protective mechanisms, including the maintenance of mitochondrial membrane potential and ATP production, while preventing reactive oxygen species (ROS) from damaging membranes. The role of antioxidant enzymes such as SOD, CAT, and GPx, as well as the activation of the Nrf2/ARE signaling pathway for cellular protection, is also depicted. Additionally, the diagram reflects how the presence of compounds like eugenol helps reduce handling‐induced oxidative stress and supports mitochondrial biogenesis, ultimately enhancing cellular resilience.

The protective effects of plant‐oil bioactives, including polyphenols and phenolic terpenoids (eugenol), on fish mitochondria during high‐intensity farming stem from three main mechanisms. The protective compounds prevent ROS from damaging membranes while maintaining lipid stability to prevent MDA formation and ferroptosis, and they protect mitochondrial membrane potential and ATP production through mPTP pore closure. The Nrf2/ARE signaling pathway starts cytoprotective responses through the activation of antioxidant enzymes SOD, CAT and GPx, which also involve SIRT1/PGC‐1α and SIRT3/mTOR pathways to support mitochondrial biogenesis and respiratory‐chain maintenance [122, 123, 125, 134, 135]. Studies about aquaculture have demonstrated that tea polyphenols and grape‐seed extracts and rosemary extracts and phytogenic essential oils from oregano and lemongrass and clove boost antioxidant enzyme activity while protecting lipids from damage and reducing the duration of recovery from handling stress. The bioavailable compound eugenol present in immersion baths protects fish from handling‐induced oxidative stress, which reduces ATP production [124, 125, 136–138]. The fish cell studies produced mechanistic results that validated the roles of Nrf2‐GPx4 and PGC‐1α/SIRT3 in preserving cellular respiration and ATP production. The recommendation requires testing across various production stages because different species exist and there is no commercial data available for ATP measurement [126, 134, 135]. The protective effects of plant‐oil bioactives on mitochondrial function in aquaculture fish are illustrated in Figure 6.

## 6. Stress and Disease Resistance

### 6.1. Environmental Stress Response and Mechanistic Pathways

The aquaculture sector faces various stress factors, from hatchery operations to the final commercial phase. Stress in fish stems from environmental factors, primarily water quality, photoperiod, oxygen status, temperature, stocking density, and feeding [139]. While stress factors directly affect fish, they also indirectly affect them by making them susceptible to disease. When stress occurs in fish, physiological mechanisms are activated, creating responses to maintain balance [103].

Stress occurs due to the secretion of adrenocorticotropic hormone (ACTH) from the anterior pituitary gland. ACTH is secreted via the hypothalamic–pituitary–adrenal axis, also known as corticotropin‐releasing hormone (CRH) [103, 140]. Stress can negatively affect many life functions in fish, such as development, reproduction, behavior, and disease resistance. At this point, vegetable oils are used to reduce stress in aquaculture thanks to their analgesic effects [10, 103, 141].

### 6.2. Pathogen Resistance

Antibiotics used against diseases in aquaculture affect the intestinal microbiota, harming beneficial microorganisms. These antibiotics lead to both the development of drug resistance and the risk of drug residue [142]. Plant‐derived products, and particularly their oils, are used as alternatives to antibiotics in combating pathogens, while also eliminating the risks of residue and resistance. Furthermore, essential oils produced from medicinal and aromatic plants stand out among natural compounds not only for their antibacterial properties but also for their antiviral, antifungal, and antiparasitic effects [57]. The antimicrobial activity of medicinal and aromatic plant oils against major aquatic pathogens is summarized in Table 2.

**Table 2:** Summary of medicinal plant essential oils, their antimicrobial activity, target pathogens, and outcomes in aquaculture species.

Essential oil (plant source)	Antimicrobial activity/main compounds	Target pathogens/species	Reported outcomes in fish/crustaceans	Tested fish/crustacean species	Experimental duration	Citations
Oregano (*Origanum vulgare*)	Strong antibacterial (carvacrol, thymol)	*Aeromonas* spp., *Vibrio* spp., *Streptococcus* spp., *Photobacterium damselae*, *Tenacibaculum maritimum*	Reduced mortality, improved immunity, growth, antioxidative status, effective in nanoemulsion and dietary forms	*Cyprinus carpio*, *Penaeus vannamei*, *Oreochromis niloticus*	8 weeks–4 days	[51, 55, 57, 58, 60, 143–146]
Thyme (*Thymus vulgaris*)	Strong antibacterial (thymol, carvacrol)	*Aeromonas* spp., *Vibrio* spp., *Streptococcus* spp., *Pseudomonas* spp.	Enhanced survival, immune response, reduced pathogen load, improved growth	*Cyprinus carpio*, *Ctenopharyngodon idellus*, *Penaeus vannamei*	4 days–8 days–60 days	[55, 57, 60, 63, 143, 145, 147, 148]
Cinnamon (*Cinnamomum* spp.)	Antibacterial, antioxidant (cinnamaldehyde)	*Aeromonas* spp., *Vibrio* spp., *Pseudomonas* spp.	Inhibited multiresistant bacteria, improved growth, antioxidant status, anesthetic effect	*Rhamdia quelen*	60 days	[63, 149, 150]
Clove (*Syzygium aromaticum*)	Antibacterial, anesthetic (eugenol)	*Aeromonas hydrophila*, *Streptococcus* spp., *Saprolegnia* spp.	Used as anesthetic, improved survival, reduced stress, moderate antibacterial	*Dicentrarchus labrax*, *Sparus aurata*, *Parapenaeus longirostris*, and *Mytilus galloprovinciali*	Not specified	[64, 67, 103, 141, 146]
Tea tree (*Melaleuca alternifolia*)	Antibacterial, antibiofilm (terpinen‐4‐ol)	*Aeromonas* spp., *Vibrio* spp., *Pseudomonas* spp.	Reduced biofilm, moderate antibacterial, improved survival	*Penaeus vannamei*	4 days	[63, 143–146]
Eucalyptus (*Eucalyptus globulus*)	Antibacterial (1,8‐cineole)	*Streptococcus* spp., *Vibrio* spp., *Aeromonas* spp.	Improved growth, immunity, gut health, moderate antibacterial	*Trachinotus ovatus*	30 days	[144, 146, 151]
Savory (*Satureja hortensis/thymbra*)	Antibacterial, antioxidant (carvacrol, p‐cymene, γ‐terpinene)	*Aeromonas* spp., *Vibrio* spp., *Bacillus* spp.	Broad‐spectrum activity, reduced biofilm, antioxidant effect	*Dicentrarchus labrax*, *Diplodus puntazzo*, *Sparus aurata*, and *Pagrus pagrus*	22 h	[57, 60, 152]
Peppermint (*Mentha piperita*)	Antiparasitic, antibacterial (menthol)	*Neoechinorhynchus buttnerae*, *Aeromonas* spp.	Effective anthelmintic, improved hematology, growth	*Colossoma macropomum*	30 days	[153]
Ginger (*Zingiber officinale*)	Antibacterial, antioxidant (geranial)	*Streptococcus agalactiae*, *Aeromonas* spp.	Improved immune response, disease resistance	*Colossoma macropomum*	30 days	[103, 153]
Lemongrass (*Cymbopogon citratus*)	Antibiofilm, antibacterial (citral)	*Vibrio parahaemolyticus*	Inhibited biofilm formation, enhanced seafood safety	*Penaeus vannamei*	4 days	[143, 154]
Cumin (*Cuminum cyminum*)	Antibiofilm, antibacterial (cuminaldehyde)	*Vibrio parahaemolyticus*	Inhibited biofilm, improved food safety	*Saurida undosquamis*, *Pagrus pagrus*, *and Mugil cephalus*	24 h	[154]
Palmarosa (*Cymbopogon martinii*)	Antiparasitic	*Gyrodactylus kobayashii*	High efficacy, safe for goldfish	*Carassius auratus*	24 h	[155]
Curcuma (*Curcuma longa*)	Antiparasitic, anesthetic	*Gyrodactylus kobayashii*	Effective, dose‐dependent, safe for goldfish	*Carassius auratus*	24 h	[155]
*Lippia alba*/origanoides	Antiparasitic, antibacterial	Monogenoideans, *Ichthyophthirius multifiliis*	In vitro efficacy, limited in vivo due to toxicity	*Colossoma macropomum*	15–60 min	[156, 157]
Bay Laurel (*Laurus nobilis*)	Antibacterial, antioxidant	*Pseudomonas* spp., *Aeromonas* spp.	Inhibited growth of spoilage/pathogenic bacteria	*Dicentrarchus labrax*, *Sparus aurata*, *Parapenaeus longirostris*, *and Mytilus galloprovincialis*	Not specified	[47, 64]
Sage, rosemary	Antibacterial, antifungal, antioxidant	*Saprolegnia* spp., *Aphanomyces astaci*	Inhibited oomycete pathogens, improved fish health	Not specified	Not specified	[47]
*Schinus terebinthifolius* (Pepper tree)	Antiparasitic, antibacterial (α‐pinene, δ‐3‐carene)	*Epistylis* sp., *Aeromonas hydrophila*, *Vibrio* spp.	100% efficacy at 2% EO, effective against multiple pathogens	*Oreochromis niloticus*	24 h	[158]
Pinaceae (*Abies holophylla*, *Pinus thunbergii*)	Antibacterial (bornyl acetate, α‐pinene)	*Edwardsiella tarda*, *Photobacterium damselae*	Strong inhibition of gram‐negative pathogens	*Paralichthys olivaceus*	24 h	[82]
Basil (*Ocimum basilicum*)	Antibiofilm, antibacterial (linalool)	*Salmonella* spp., *Bacillus* spp.	Reduced biofilm, effective against MDR strains	*Oncorhynchus mykiss*	24 h	[159]

Antibiotics used against diseases in aquaculture affect the intestinal microbiota, harming beneficial microorganisms. These antibiotics lead to both the development of drug resistance and the risk of drug residue [142]. Plant‐derived products, and particularly their oils, are used as alternatives to antibiotics in combating pathogens, while also eliminating the risks of residue and resistance. Furthermore, essential oils produced from medicinal and aromatic plants stand out among natural compounds not only for their antibacterial properties but also for their antiviral, antifungal, and antiparasitic effects [57].

Medicinal and aromatic plants and their oils have an antibacterial effect on bacteria by stopping their growth or killing bacterial cells by disintegration [160].

It has been reported that plant species such as *Origanum onites*, *Thymbra spicata*, and *Cinnamomum zeylanicum/verum* have antibacterial properties on *Aeromonas salmonicida; Cymbopogon citratus*, and *Thymus vulgaris* on *Vibrio* spp.; *Ocimum basilicum*, *T. vulgaris*, and *Laurus nobilis* on *Pseudomonas* spp.; and *C. nardus* on *Flavobacterium* spp [143, 161–163].

Vegetable oils target the cell walls of fungal cells. They disrupt the function of the cell wall by creating a membrane potential. While cell membrane function remains intact, ATP formation is inhibited. Due to the loss of cell membrane function, certain changes occur in permeability processes. These changes disrupt the fungal cell wall and protoplasm, resulting in an antifungal effect [160]. Studies have shown the antifungal activity of clove and thyme oils against *Saprolegnia* sp. fungi in fish [164, 165].

Plant‐based products are considered a new‐generation alternative antiviral agent due to their high genetic chemical diversity. Clove and thyme oils exhibit high antiviral properties against many nonenveloped RNA and DNA viruses [160].

For example, antiviral activity against white spot syndrome virus (WSSV) has been reported in fish from *Gardenia jasminoide*, *Olea europaea*, and *Argemone mexicana*. Coumarin derivatives also have antiviral activity against spring viremia of carp virus (SVCV). Furthermore, *Clinacanthus nutans* exhibits antiviral activity against *Cyprinid herpesvirus 3* (*CyHV-3*) [166].

**Figure 7:**
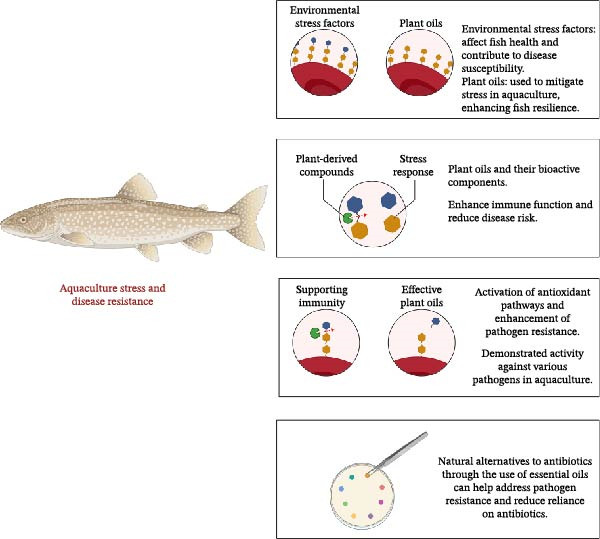
Overview of stress and disease resistance mechanisms in aquaculture fish. The diagram highlights environmental stress factors that affect fish health and enhance susceptibility to disease. It illustrates the use of plant‐derived compounds, particularly essential oils, to mitigate stress and support immune function. The activation of antioxidant pathways and the demonstrated efficacy of natural alternatives to antibiotics against various pathogens in aquaculture are also depicted, underscoring the potential benefits of these approaches in enhancing fish resilience.

The antiparasitic efficacy of many herbal products has also been evaluated. According to these evaluations, the antiparasitic activities of *Piper* genus herbal products against *Neoechinorhynchus buttnerae*, *Lippia* genus plants against *Dactylogyrus* spp., and *Varronia curassavica* oil against *Ichthyophthirius multifiliis* have been proven [31, 142, 167, 168]. Aquaculture stress factors and their impacts on disease resistance, along with the role of plant‐derived compounds in mitigating these effects, are illustrated in Figure 7.

## 7. Practical Applications in Aquafeeds

### 7.1. Optimal Inclusion Levels

Medicinal and aromatic plants present many challenges when used as feed additives. Due to the chemical structure of the plants, the dose–response relationship is quite delicate. For example, while thyme and tamarind leaves are highly beneficial, these benefits are highly dependent on dosing [169].

In addition, the proportion of vegetable oils in the diet varies depending on the plant species and fish species. For example, in carp, the proportion of vegetable oils is generally effective when used at 0.5%–0.75% of the diet, while neroli oil is effective at 0.25%–0.50% of the diet [170]. In another study, the effects of anise oil and cumin oil on growth and antioxidant parameters in Nile tilapia (*Oreochromis niloticus*) were different in the proportions of the diets [44, 171]. Reported dietary inclusion levels of medicinal and aromatic plant oils and their dose‑dependent effects on growth performance and health parameters in different fish species are summarized in Table 3.

**Table 3:** Dietary inclusion levels of medicinal and aromatic plant oils in aquafeeds and their dose‑dependent effects on fish growth and health.

Fish species	Oil type (plant source)	Dose range (per kg feed)	Observed effects on growth and health	Experimental duration	Consistency/level of evidence	Citations
Common carp (*Cyprinus carpio*)	Oregano essential oil (*Origanum vulgare*)	0–20 g (optimum: 15 g)	Improved antioxidative status, immune gene expression, survival post‐infection; best at 15 g/kg	8 weeks	Positive/neutral	[51, 52, 170, 172, 173]
*Channa punctata*	*Curcuma aromatica* essential oil	0–2 mL (optimum: 2 mL)	Enhanced growth, immunity, survival (>95%) after bacterial challenge	7 weeks	Positive	[72]
European seabass (*Dicentrarchus labrax*)	Lemongrass, Chamomile essential oils	2 mL	Improved growth, feed utilization, protein levels, reduced stress, lower bacterial load	30 days	Neutral/negative	[174–176]
Nile tilapia (*Oreochromis niloticus*)	Herbal essential oil blend (carvacrol, oregano, thymol, etc.)	0–240 mL (optimum: 30–60 mL)	Enhanced growth, immunity, antioxidant status at 30–60 mL/kg; higher doses detrimental	72 days	Negative/toxic (at high doses)	[58, 69, 78, 177–185]
Common carp (*Cyprinus carpio*)	Plant‐based feed additives (various EOs)	0.04%–2% (EOs: 0.5%–0.75%)	Improved feed efficiency, growth, immunity at 0.5%–0.75% EO; higher doses may suppress growth	Not specified	Dose‐dependent	[170]
Hybrid red tilapia (*Oreochromis* spp.)	Oregano essential oil	0–1.2 g (optimum: 1.2 g)	Improved growth, survival, feed efficiency, gut morphology, reproductive indices	13 weeks	Strong positive	[59]
Rainbow trout (*Oncorhynchus mykiss*)	Oregano essential oil (*O. onites*)	0.125–3.0 mL (optimum: 3.0 mL)	Enhanced growth, feed conversion, antioxidant status, lysozyme activity, disease resistance	90 days	Neutral	[52, 54, 186]
African catfish (*Clarias gariepinus*)	Clove and peppermint oil blend	0–3 mL (optimum: 1–2 mL)	Improved growth, feed utilization, immunity, antioxidant status, organ histology	2 months	Positive	[83]
Caspian roach (*Rutilus caspicus*)	Savory essential oil (*Satureja hortensis*)	0–400 mg (optimum: 200 mg)	Best growth, antioxidant, immune parameters, and salinity stress resistance at 200 mg/kg	60 days	Negative (at highest dose)	[187]
Channel catfish (*Ictalurus punctatus*)	*Litsea cubeba* essential oil	0–800 mg (optimum: 100–200 mg)	Improved growth, immunity, intestinal health, beneficial microbiota at 100–200 mg/kg	Not specified	Positive	[188]
Tambaqui (*Colossoma macropomum*)	Lippia sidoides essential oil	0–1.5 mL (optimum: 0.5–1.0 mL)	Improved growth, metabolism, hematology, digestive enzymes at 0.5–1.0 mL/kg	60 days	Negative (at high doses)	[189]
Neotropical catfish (*Lophiosilurus alexandri*)	Oregano essential oil	0–4 g (optimum: 2.1–2.56 g)	Maximized growth, protein retention, muscle hyperplasia at 2.1–2.56 g/kg	13 weeks	Neutral	[190]
Red swamp crayfish (*Procambarus clarkii*)	Oregano essential oil	0–500 mg (optimum: 500 mg)	Enhanced antioxidant and immune gene expression, no negative tissue effects	21 days	Positive	[191]
*Trachinotus ovatus*	Eucalyptus essential oil	0–15 mL (optimum: 5–10 mL)	Improved growth, gut morphology, immune response, reduced pathogenic bacteria at 5–10 mL/kg	30 days	Negative (at highest dose)	[151]
Tambatinga (*Colossoma* x *Piaractus*)	*Melaleuca alternifolia* essential oil	0–2.5 mL (optimum: >1.5 mL)	No growth effect, but increased survival and resistance to *Saprolegnia* at >1.5 mL/kg	45 days	Neutral/positive	[192]
Nile tilapia (*Oreochromis niloticus*)	Ginger essential oil (*Zingiber officinale*)	0–2 mL (optimum: 0.5 mL)	Improved growth, feed conversion, hematology at 0.5 mL/kg; higher doses caused liver dysfunction	60 days	Negative/toxic (at high doses)	[177]
Nile tilapia (*Oreochromis niloticus*)	Anise oil (*Pimpinella anisum*)	0%–0.3% (optimum: 0.1%–0.2%)	Improved growth, antioxidant status at 0.1%–0.2%; 0.3% caused adverse effects	8 weeks	Negative/toxic (at high dose)	[44]
Nile tilapia (*Oreochromis niloticus*)	*Santalum album* essential oil	0–4 mL (optimum: 2 mL)	Enhanced immunity, antioxidant status, survival against *S. aureus* at 2 mL/kg	60 days	Negative (at high doses)	[183]
Nile tilapia (*Oreochromis niloticus*)	Caraway essential oil	0–0.5 g (optimum: 0.1 g)	Improved growth, feed utilization, muscle quality at 0.1 g/kg	12 weeks	Positive	[178]
Nile tilapia (*Oreochromis niloticus*)	*Aloysia triphylla* essential oil	0–2 mL (optimum: 2 mL)	Improved growth, hematology, digestive enzymes, lysozyme activity at 2 mL/kg	Not specified	Positive	[179]
Nile tilapia (*Oreochromis niloticus*)	Lippia sidoides essential oil	0%–1.25% (optimum: 0.25%)	Improved organ integrity, antioxidant enzyme activity at 0.25%	15 days	Negative/toxic (at high doses)	[193]
Nile tilapia (*Oreochromis niloticus*)	Thyme essential oil (*Thymus vulgaris*)	0%–2% (optimum: 0.5%–1%)	Enhanced growth, immunity, disease resistance at 0.5%–1%	28–60 days	Neutral/negative (at high doses)	[186, 194]
Nile tilapia (*Oreochromis niloticus*)	Oregano essential oil (salinized water)	0–3 g (optimum: 0.75–3 g)	Best growth at 0.75 g/kg; 3 g/kg improved intestinal villi	Not specified	Positive	[180]
Nile tilapia (*Oreochromis niloticus*)	Orange/lemon essential oil blend	0–3‰ (optimum: 3‰ OEO + 1‰ LEO)	Improved growth, immunity, antioxidant status, gut morphology at highest blend dose	60 days	Strong positive	[184]
Nile tilapia (*Oreochromis niloticus*)	Essential oil blend (carvacrol, thymol, cinnamaldehyde)	0–10 g (optimum: 3.3 g)	Improved growth, modulated gut microbiota at 3.3 g/kg	60 days	Neutral	[94]
Nile tilapia (*Oreochromis niloticus*)	Essential oil mix (cashew, castor bean)	0%–0.2% (no effect on growth)	No growth effect, but improved HDL/LDL at >0.15%	60 days	Neutral	[195]
Tambaqui (*Colossoma macropomum*)	Lippia grata essential oil	0–2 mL (optimum: 0.5 mL)	Improved carcass yield at all doses; best growth at 0.5 mL/kg	60 days	Negative/toxic (at high doses)	[196]

It is crucial to note that the biological responses to MAPOs are not uniformly positive, and several interpretations of their mechanisms remain hypothesis‐based. Literature reveals frequent species‐specific variability, with several studies reporting neutral or even negative outcomes depending on the dosage and formulation. For instance, while optimal doses improve growth and immunity, higher inclusion levels often lead to growth suppression, liver dysfunction, or intestinal damage due to the potential toxicity of concentrated volatile compounds [170, 177]. Furthermore, in some trials, specific essential oils yielded no significant effect on growth performance, functioning only as mild immunostimulants or exhibiting no metabolic benefits compared to control diets [192, 195]. These conflicting findings highlight that proposed mechanistic pathways (e.g., microbiome regulation or endocrine modulation) must be interpreted cautiously, necessitating rigorous, molecular‐level validation before widespread commercial adoption.

### 7.2. Synergistic Combinations

The use of medicinal and aromatic plant oils combined with various additives is common in the diet. In this regard, probiotics, prebiotics, organic acids, and enzymes, in particular, offer significant benefits through their synergistic effects. Probiotics offer advantages, particularly through their benefits on the microbiota, while organic acids contribute to intestinal health by reducing pH levels, supporting both the microbiota and acting on pathogenic agents [197, 198]. Enzymes added to the diet support nutrient digestion by eliminating antinutritional factors and supporting endogenous enzymes [198]. Their combined use, combined with their antioxidant, antimicrobial, and immunostimulatory properties, offers significant protective potential in living organisms. Beyond organic and microbial additives, the strategic combination of MAPOs with functional trace elements represents a highly promising and underexplored frontier. For example, recent findings highlight that dietary supplementation with specific trace minerals, such as boric acid, significantly enhances growth performance, improves hematological profiles, and fortifies the antioxidant defense system (by reducing malondialdehyde levels and modulating SOD and CAT activities) in salmonids like rainbow trout [199]. Complementary studies on Nile tilapia have also established the LC_50_ values for boron and confirmed that low‐level exposure, whether through water or feed, can significantly influence growth metrics and essential hematological parameters, such as red blood cell counts and hemoglobin levels. These findings further support the integration of such trace elements into functional aquafeed strategies to enhance physiological stability in diverse aquaculture species [200].

Co‐supplementing these oxidative‐stress‐relieving minerals with bioactive plant oils could create powerful synergistic feed formulations to maximize physiological resilience in intensive aquaculture.

Studies on the combined use of probiotics and herbs have shown improved growth performance in a variety of aquaculture species, from freshwater to saltwater fish, and in crustaceans. Furthermore, combined use with probiotics has been shown to support the immune system and increase resistance to pathogens [201].

Similarly, another study focused on the combined use of a multi‐enzyme complex, organic acid, an essential oil complex, and prebiotics and reported that their combined use positively affected growth performance and feed utilization in rainbow trout (*Oncorhynchus mykiss*) [202].

### 7.3. Advanced Delivery Systems

Vegetable oils are hydrophobic natural products with very low water solubility. Essential oils, vital for aromatic and medicinal plants, contain metabolites such as monoterpenes and phenylpropenes. Due to their volatile and degradable properties, vegetable oils are quite difficult to use in a controlled and efficient manner. Their low solubility in water further complicates their use as a dietary food [203, 204]. Therefore, advanced systems are being used to utilize vegetable oils more efficiently.

Emulsion systems are a preferred method for more efficient use of vegetable oils. Nanoemulsions are one of the most effective methods for delivering the bioactive components of vegetable oils thanks to their nanometric droplet‐sized encapsulation properties. Based on the use of nonionic surfactants, nanoemulsions composed of different plant‐based oils are highly safe, highly stable, and highly biocompatible [205, 206].

Additionally, nanotechnologies are also being used in drug delivery systems to provide drugs with longer transport times. Alginate and chitosan are attracting significant interest in this regard due to their biocompatibility, long circulation time, and biodegradability. Nanoencapsulation of herbal products with alginate‐chitosan combinations has been reported to positively impact immune and antioxidant systems [207]. Despite the promising in vitro and small‐scale in vivo results of these advanced delivery systems, their transition to commercial aquafeed manufacturing faces significant practical hurdles. Traditional aquafeed production relies heavily on extrusion processes, which involve high temperatures (often exceeding 100°C), high pressure, and severe mechanical shear. These harsh conditions can easily degrade volatile terpenes, rupture microcapsules, and destabilize lipid‐based nanocarriers if they are not thermally robust. Furthermore, the cost‐effectiveness and scalability of nanoencapsulation remain major bottlenecks for industry adoption. Utilizing specialized biopolymers (like purified chitosan or alginate) and advanced homogenization equipment significantly increases the unit cost of the final feed. For these technologies to be commercially viable, future research and feed engineering must focus on developing affordable, food‐grade encapsulants and rigorously validating the thermal stability and retention rates of MAPOs during industrial‐scale extrusion.

### 7.4. Challenges and Solutions

Medicinal and aromatic plant oils exhibit beneficial biological activities in many ways. However, despite these positive effects, they present significant challenges in their use due to potential drawbacks during their extraction and storage. These challenges generally arise from oxidation, degradation, polymerization, and isomerization, which occur due to environmental factors (temperature, oxygen, and light) [208].

**Figure 8:**
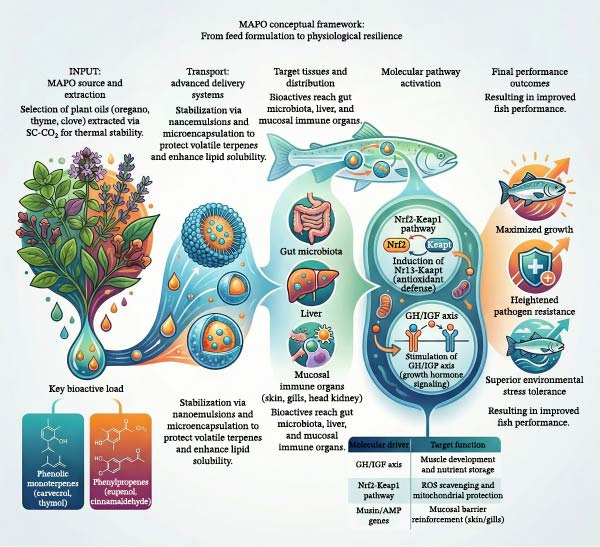
Comprehensive conceptual framework integrating the application of medicinal and aromatic plant oils (MAPOs) in aquaculture. The flowchart illustrates the progression from plant‐source extraction and advanced formulation strategies (e.g., nanoemulsions, liposomes) to targeted physiological systems (gut microbiota, liver, immune organs), specific molecular pathways (GH/IGF, Nrf2‐Keap1), and the ultimate biological outcomes encompassing growth promotion, disease resistance, and stress resilience.

Due to these challenges, vegetable oils can partially or completely lose their effectiveness. Furthermore, due to their physical and chemical structure, vegetable oils exhibit a weak structure with short half‐lives and high volatility. Considering all these factors, nanotechnologies are important in addressing many challenges, considering their potential for transporting vegetable oils, improving their physical and chemical properties, increasing their stability, improving their water solubility, minimizing their volatility, and providing protection against environmental interactions [209, 210]. To synthesize the multifactorial mechanisms discussed throughout this review, a comprehensive conceptual framework integrating MAPO extraction, delivery technologies, physiological targets, molecular pathways, and ultimate biological outcomes is presented in Figure 8.

## 8. Sustainability and Future Perspectives

### 8.1. MAPOs as Antibiotic Alternatives

Plant‐oil (essential oil/extract) supplementation shows immunostimulatory and antioxidant effects, as well as in vivo and in vitro antimicrobial and antibiofilm properties, which can contribute to lowering disease incidence as a preventive measure. However, plant oils rarely match the rapid, high‐potency bactericidal action of therapeutic antibiotics in acute outbreaks [10, 142, 211]. The products present themselves as low‐impact preventive solutions that could be affordable, yet researchers lack sufficient data to compare their costs against antibiotic treatments [212–214]. Regulatory acceptance varies significantly across global markets. For instance, while the EU has banned antibiotic growth promoters, plant oils must still navigate rigorous feed‐additive or veterinary approval processes. These pathways require extensive safety data and efficacy validation to obtain market authorization [215–217]. The acceptance of these solutions by consumers depends on their antibiotic‐free status and their sensory qualities and their shelf life and their ability to provide accurate labeling and tracking information [218–220].

### 8.2. Circular Economy and Plant By‐Products

The waste materials from agricultural operations and food processing (basil extraction wastes and citrus peels and spice residues) can be transformed into valuable products through essential oil extraction, which results in aquafeed ingredients that serve as immunostimulants and antioxidants and antimicrobial adjuvants. Research shows that plant oils used in fish feed produce health advantages and stable feed characteristics, which make them suitable substitutes for synthetic feed additives [29, 45, 142]. The entire product life cycle benefits from waste diversion programs and synthetic additive reduction and antibiotic reduction and antibiotic residue minimization strategies. Research shows that residue conversion into new feed materials and essential oil applications for AMR control can decrease environmental impacts from feed production and eutrophication risks according to One Health frameworks [142, 217]. The use of regionally sourced oils helps reduce transportation emissions while building stable supply networks that support circular economic systems throughout local communities [221, 222]. The economic advantages of locating extraction facilities near processing facilities allow businesses to create multiple revenue streams while they improve operational efficiency and meet customer demands for antibiotic‐free products. Companies need to invest money for nanoemulsion development because they must conduct research on extraction methods and formulation techniques to achieve better stability and effectiveness [29, 50].

### 8.3. Genomics‐Based Precision Nutrition and AI‐Driven Formulations

Research using SNPs/QTLs and population genomics enables scientists to detect fish population responses to phytogenic oils through sea bass studies, which reveal particular genetic reactions to phytogenic compounds. Scientists use these interactions to create dosing methods and breeding programs that reduce phytogenic response variations between different fish individuals [223]. While the current practical application of these advanced technologies in commercial aquaculture is still in its early stages, primarily limited to basic genetic screening for trait selection and early‐stage AI models for disease diagnosis and management [217], the future perspective is highly promising. Moving forward, it is theoretically envisioned that AI and machine learning (ML) pipelines will be able to integrate host genomic data, microbiome profiles, pathogen prevalence, and specific MAPO chemotypes. From a future perspective, such integration could predict optimal, farm‐specific blend combinations and precise dosages. By adapting established methods from human gene‐diet modeling, future *in silico* formulation capabilities could eventually minimize the trial‐and‐error phase in aquafeed development, bridging the gap between molecular biology and practical, large‐scale feed manufacturing [224]. Research shows that plants that obtain their nutrients through genetic inheritance develop stronger immune systems and higher antioxidant levels while showing reduced disease occurrence and needing less antibiotic treatment, which leads to better feed efficiency and reduced environmental damage from antibiotic waste and suboptimal feeding practices [214, 223]. The implementation of GBS/microsatellite pipelines needs standardized methods to process various genotyping approaches and chemotype definitions and restricted farm information and limited AMR monitoring based on genomic and AMR research guidelines [29, 225, 226].

## 9. Conclusion

MAPOs represent a rapidly evolving class of functional aquafeed additives capable of addressing key challenges introduced by the transition from marine ingredients to plant‐, insect‐, and microbial‐based diets. While responses can exhibit significant heterogeneity depending on the fish species, dosage, and environmental factors, phenolic monoterpenes, phenylpropenes, and related terpenoids have frequently been shown to stimulate appetite, enhance digestive enzyme secretion, and modulate gut microbiota composition, potentially converging on growth‐regulatory endocrine axes such as the GH/IGF system. Parallel to these nutritional benefits, MAPOs activate antioxidant defenses through Nrf2‐Keap1 signaling, stabilize mitochondrial function, and reinforce mucosal and systemic immunological pathways. These multifactorial responses position MAPOs as biologically active agents that do more than compensate for the absence of FM and O; they enable metabolic resilience, disease resistance, and performance stability in intensive aquaculture.

Despite this promise, MAPO research remains fragmented by major operational and methodological limitations. Essential oil chemotypes vary with genotype, environment, harvest timing, and extraction technology, yet most studies provide minimal compositional metadata, hindering reproducibility and cross‐study comparisons. Dose–response relationships are frequently explored within narrow experimental windows, while immune or antioxidant endpoints are inconsistently measured across tissues and developmental stages. Delivery methods also remain underdeveloped: direct inclusion of volatile oils often leads to oxidative loss, intestinal irritation, or inconsistent bioavailability, whereas nanoemulsions, microencapsulation, and lipid carriers, though methodologically superior, are rarely validated under farm‐scale conditions. These gaps collectively impede the translation of MAPOs from trial‐level outcomes to standardized, commercially deployable feed formulations.

Future progress requires aligning mechanistic research with industrial realities. Chemotype‐standardized MAPOs should be profiled through GC–MS/HPLC fingerprinting and integrated into dose–response studies that capture endocrine signaling, microbiome remodeling, oxidative stress kinetics, and mucosal barriers across species and life stages. Omics‐driven approaches, metatranscriptomics, metabolomics, lipidomics, and host genomics will be essential to map the interactions between MAPO molecules, host tissues, and microbial consortia and to identify biomarkers of efficacy and tolerance. Equally important is evaluating MAPOs within multi‐additive systems, including probiotics, enzymes, and organic acids, where synergistic performance effects may exceed that of individual components. These scientific advances must be supported by regulatory frameworks that recognize MAPOs as functional feed additives, not just flavoring agents, and by commercial strategies that leverage circular economy principles to valorize agricultural by‐products as phytogenic feed resources.

Ultimately, MAPOs will not replace therapeutic antibiotics in acute outbreaks, but their preventive capacity—strengthening immunity, reducing oxidative stress, modulating microbiota, and supporting physiological resilience—provides a sustainable cornerstone for next‐generation aquafeeds. By integrating chemotype standardization, advanced delivery technologies, omics‐based precision nutrition, and real‐world farm validation, MAPOs can evolve from promising laboratory supplements into reliable tools that enhance animal health, feed efficiency, and environmental sustainability in modern aquaculture. For immediate industry application, a phased approach is recommended: feed manufacturers should initially focus on well‐characterized MAPO blends (e.g., oregano and thyme derivatives) at low, strictly monitored inclusion levels as preventive health boosters while conducting parallel on‐farm trials to evaluate their stability during the extrusion process. Future experimental priorities must pivot from short‐term, controlled laboratory trials to longitudinal, commercial‐scale studies. These studies should explicitly evaluate the cost–benefit ratio, thermal stability of advanced delivery systems (such as nanoemulsions), and long‐term impacts on fish health to bridge the gap between academic research and industrial‐scale aquaculture.

## Author Contributions

Mustafa Öz conceptualized and supervised the study. All authors contributed equally to the literature review, data interpretation, and manuscript preparation.

## Funding

This research received no specific grant from any funding agency in the public, commercial, or not‐for‐profit sectors.

## Disclosure

Each author participated in drafting, reviewing, and approving the final version of the manuscript and agreed to be accountable for all aspects of the work.

## Ethics Statement

This article does not contain any studies with human participants or animals performed by any of the authors.

## Conflicts of Interest

The authors declare no conflicts of interest.

## Data Availability

All data and materials used in this review are drawn from published sources and properly cited in the manuscript. No new datasets were generated or analyzed during the current study.

## References

[1] Fernando I. , Nisa’ F. , and Yudhistira B. , et al.A Comprehensive Review on the Utilization of Housefly Larvae (*Musca domestica*) as Dietary Meals in Aquaculture, Discover Sustainability. (2025) 6, no. 1, 10.1007/s43621-025-01826-1, 986.

[2] McKuin B. , Kapuscinski A. R. , Sarker P. K. , Cheek N. , Lim J. , and Sabarsky M. , Comparative Life Cycle Assessment of Marine Microalgae, *Nannochloropsis* sp. and Fishmeal for Sustainable Protein Ingredients in Aquaculture Feeds, Elem Sci Anth. (2023) 11, no. 1, 10.1525/elementa.2022.00083, 00083.

[3] Meetei K. B. , Nongmaithem B. D. , and Ngangbam A. K. , Aquafeed Innovation: Utilization of Molluscan and Crustacean By-Products for Sustainable Fish Nutrition, Journal of Experimental Agriculture International. (2025) 47, no. 3, 349–356, 10.9734/jeai/2025/v47i33341.

[4] Öz M. and İnanan B. E. , The Role of Glutamine in Growth, Health and Sensory Quality of Female Rainbow Trout (*Oncorhynchus mykiss*), Veterinary Medicine and Science. (2025) 11, no. 5, 10.1002/vms3.70527, e70527. 40726044 PMC12304083

[5] Ahmed S. R. , Hoseinifar S. H. , and Van Doan H. , et al.Implications of Fermented Plant Protein Ingredients: A Critical Review of Nutrition, Physiology and Growth Related Aspects, Annals of Animal Science. (2025) 25, no. 4, 1345–1360, 10.2478/aoas-2025-0010.

[6] Caballero-Solares A. , Eslamloo K. , and Hall J. R. , et al.Vegetable Omega-3 and Omega-6 Fatty Acids Differentially Modulate the Antiviral and Antibacterial Immune Responses of Atlantic Salmon, Scientific Reports. (2024) 14, no. 1, 10.1038/s41598-024-61144-w, 10947. 38740811 PMC11091188

[7] Siddik M. A. B. , Julien B. B. , Islam S. M. M. , and Francis D. S. , Fermentation in Aquafeed Processing: Achieving Sustainability in Feeds for Global Aquaculture Production, Reviews in Aquaculture. (2024) 16, no. 3, 1244–1265, 10.1111/raq.12894.

[8] Hashem N. M. , Hosny N. S. , El-Desoky N. I. , and Shehata M. G. , Effect of Nanoencapsulated Alginate-Synbiotic on Gut Microflora Balance, Immunity, and Growth Performance of Growing Rabbits, Polymers. (2021) 13, no. 23, 10.3390/polym13234191, 4191. 34883694 PMC8659830

[9] Nakano T. and Wiegertjes G. , Properties of Carotenoids in Fish Fitness: A Review, Marine Drugs. (2020) 18, no. 11, 10.3390/md18110568, 568. 33227976 PMC7699198

[10] Sutili F. J. , Gatlin D. M.III, Heinzmann B. M. , and Baldisserotto B. , Plant Essential Oils as Fish Diet Additives: Benefits on Fish Health and Stability in Feed, Reviews in Aquaculture. (2018) 10, no. 3, 716–726, 10.1111/raq.12197, 2-s2.0-85017402197.

[11] Tompkins E. , Cadieux B. , Amitrano M. , and Goodridge L. , High-Throughput Screening of Natural Compounds for Prophage Induction in Controlling Pathogenic Bacteria in Food, Frontiers in Food Science and Technology. (2023) 3, 10.3389/frfst.2023.1239884, 1239884.

[12] Amenyogbe E. , Droepenu E. K. , and Ayisi C. L. , et al.Impact of Probiotics, Prebiotics, and Synbiotics on Digestive Enzymes, Oxidative Stress, and Antioxidant Defense in Fish Farming: Current Insights and Future Perspectives, Frontiers in Marine Science. (2024) 11, 10.3389/fmars.2024.1368436, 1368436.

[13] Dawood M. A. O. , Koshio S. , and Esteban M. , Beneficial Roles of Feed Additives as Immunostimulants in Aquaculture: A Review, Reviews in Aquaculture. (2018) 10, no. 4, 950–974, 10.1111/raq.12209, 2-s2.0-85027490656.

[14] Puri P. , Sharma J. G. , and Singh R. , Biotherapeutic Microbial Supplementation for Ameliorating Fish Health: Developing Trends in Probiotics, Prebiotics, and Synbiotics use in Finfish Aquaculture, Animal Health Research Reviews. (2022) 23, no. 2, 113–135, 10.1017/S1466252321000165. 36597760

[15] Ahmadifar E. , Yousefi M. , and Karimi M. , et al.Benefits of Dietary Polyphenols and Polyphenol-Rich Additives to Aquatic Animal Health: An Overview, Reviews in Fisheries Science & Aquaculture. (2021) 29, no. 4, 478–511, 10.1080/23308249.2020.1818689.

[16] Ahmadifar E. , Mohammadzadeh S. , and Kalhor N. , et al.Cornelian Cherry (*Cornus mas* L.) Fruit Extract Improves Growth Performance, Disease Resistance, and Serum Immune-and Antioxidant-Related Gene Expression of Common Carp (*Cyprinus carpio*), Aquaculture. (2022) 558, 10.1016/j.aquaculture.2022.738372, 738372.

[17] Mehrinakhi Z. , Ahmadifar E. , Sheikhzadeh N. , Moghadam M. S. , and Dawood M. A. O. , Extract of Grape Seed Enhances the Growth Performance, Humoral and Mucosal Immunity, and Resistance of Common Carp (*Cyprinus carpio*) Against *Aeromonas hydrophila* , Annals of Animal Science. (2021) 21, no. 1, 217–232, 10.2478/aoas-2020-0049.

[18] Ahmadifar E. , Kalhor N. , and Yousefi M. , et al.Effects of Dietary *Plantago ovata* Seed Extract Administration on Growth Performance and Immune Function of Common Carp (*Cyprinus carpio*) Fingerling Exposed to Ammonia Toxicity, Veterinary Research Communications. (2023) 47, no. 2, 731–744, 10.1007/s11259-022-10034-5. 36400970

[19] Ahmadifar E. , Sheikhzadeh N. , Roshanaei K. , Dargahi N. , and Faggio C. , Can Dietary Ginger (*Zingiber officinale*) Alter Biochemical and Immunological Parameters and Gene Expression Related to Growth, Immunity and Antioxidant System in Zebrafish (*Danio rerio*)?, Aquaculture. (2019) 507, 341–348, 10.1016/j.aquaculture.2019.04.049, 2-s2.0-85064825469.

[20] Van Doan H. , Lumsangkul C. , and Sringarm K. , et al.Impacts of Amla (*Phyllanthus emblica*) Fruit Extract on Growth, Skin Mucosal and Serum Immunities, and Disease Resistance of Nile Tilapia (*Oreochromis niloticus*) Raised Under Biofloc System, Aquaculture Reports. (2022) 22, 10.1016/j.aqrep.2021.100953, 100953.

[21] Harikrishnan R. , Devi G. , and Van Doan H. , et al.Dietary Plant Pigment on Blood-Digestive Physiology, Antioxidant-Immune Response, and Inflammatory Gene Transcriptional Regulation in Spotted Snakehead (*Channa punctata*) Infected With *Pseudomonas aeruginosa* , Fish & Shellfish Immunology. (2022) 120, 716–736, 10.1016/j.fsi.2021.12.033. 34968713

[22] Dokou S. , Bitchava K. , and Stylianaki I. , et al.Nano-Oregano Essential Oil Improves Rainbow Trout’s (*Oncorhynchus mykiss*) Growth Performance, Oxidative Status, Fatty Acid Profile of Fillet, Affects Gene Expression and Supports Skin and Intestinal Histomorphometry, Annals of Animal Science. (2023) 23, no. 4, 1177–1189, 10.2478/aoas-2023-0032.

[23] Firmino J. P. , Vallejos-Vidal E. , and Balebona M. C. , et al.Diet, Immunity, and Microbiota Interactions: An Integrative Analysis of the Intestine Transcriptional Response and Microbiota Modulation in Gilthead Seabream (*Sparus aurata*) Fed an Essential Oils-Based Functional Diet, Frontiers in Immunology. (2021) 12, 10.3389/fimmu.2021.625297, 625297. 33746962 PMC7969985

[24] Aziz E. E. , Badawy E. M. , Zheljazkov V. D. , Nicola S. M. , and Fouad H. , Yield and Chemical Composition of Essential Oil of *Achillea millefolium* L. as Affected by Harvest Time, Egyptian Journal of Chemistry. (2019) 62, no. 3, 533–540.

[25] Sgarbossa J. , Schmidt D. , Schwerz F. , Schwerz L. , Prochnow D. , and Caron B. O. , Effect of Season and Irrigation on the Chemical Composition of *Aloysia triphylla* Essential Oil, Revista Ceres. (2019) 66, no. 2, 85–93, 10.1590/0034-737x201966020002, 2-s2.0-85067016383.

[26] ds Silva T. L. M. , ds Rosa G. I. , and dos Santos M. A. L. , et al.Lemongrass Essential Oil (*Cymbopogon citratus* (DC) Stapf.) Seasonal Evaluation and Microencapsulation by Spray-Drying, Brazilian Archives of Biology and Technology. (2023) 66, no. spe, 10.1590/1678-4324-ssbfar-2023230016, e23230016.

[27] Barbas L. A. L. , Maltez L. C. , and Stringhetta G. R. , et al.Properties of Two Plant Extractives as Anaesthetics and Antioxidants for Juvenile Tambaqui *Colossoma macropomum* , Aquaculture. (2017) 469, 79–87, 10.1016/j.aquaculture.2016.12.012, 2-s2.0-85004010192.

[28] Muhammad S. , H. P. S. A. K. , and Abd Hamid S. , et al.Characterization of Bioactive Compounds From Patchouli Extracted via Supercritical Carbon Dioxide (SC-CO2) Extraction, Molecules. (2022) 27, no. 18, 10.3390/molecules27186025, 6025. 36144760 PMC9503852

[29] Reverter M. , Tapissier-Bontemps N. , Sasal P. , and Saulnier D. , Use of Medicinal Plants in Aquaculture, Diagnosis and Control of Diseases of Fish and Shellfish, 2017, 223–261.

[30] Bag A. and Chattopadhyay R. R. , Evaluation of Antioxidant Potential of Essential Oils of Some Commonly Used Indian Spices in In Vitro Models and in Food Supplements Enriched With Omega-6 and Omega-3 Fatty Acids, Environmental Science and Pollution Research. (2018) 25, no. 1, 388–398, 10.1007/s11356-017-0420-5, 2-s2.0-85031922488. 29039041

[31] Costa M. F. , Durço A. O. , Rabelo T. K. , Barreto R. S. S. , and Guimarães A. G. , Effects of Carvacrol, Thymol and Essential Oils Containing Such Monoterpenes on Wound Healing: A Systematic Review, Journal of Pharmacy and Pharmacology. (2019) 71, no. 2, 141–155, 10.1111/jphp.13054, 2-s2.0-85058187276. 30537169

[32] Gutiérrez-del-Río I. , López-Ibáñez S. , and Magadán-Corpas P. , et al.Terpenoids and Polyphenols as Natural Antioxidant Agents in Food Preservation, Antioxidants. (2021) 10, no. 8, 10.3390/antiox10081264, 1264. 34439512 PMC8389302

[33] Modareskia M. , Fattahi M. , and Mirjalili M. H. , Thymol Screening, Phenolic Contents, Antioxidant and Antibacterial Activities of Iranian Populations of *Trachyspermum ammi* (L.) Sprague (Apiaceae), Scientific Reports. (2022) 12, no. 1, 10.1038/s41598-022-19594-7, 15645. 36123425 PMC9485261

[34] Liu S. , Zhao C. , and Cao Y. , et al.Comparison of Chemical Compositions and Antioxidant Activity of Essential Oils From *Litsea Cubeba*, Cinnamon, Anise, and Eucalyptus, Molecules. (2023) 28, no. 13, 10.3390/molecules28135051, 5051. 37446712 PMC10343171

[35] Daeihamed M. , Dadashzadeh S. , Haeri A. , and Akhlaghi M. F. , Potential of Liposomes for Enhancement of Oral Drug Absorption, Current Drug Delivery. (2017) 14, no. 2, 289–303, 10.2174/1567201813666160115125756, 2-s2.0-85013900747. 26768542

[36] Hsu C.-Y. , Wang P.-W. , Alalaiwe A. , Lin Z.-C. , and Fang J.-Y. , Use of Lipid Nanocarriers to Improve Oral Delivery of Vitamins, Nutrients. (2019) 11, no. 1, 10.3390/nu11010068, 2-s2.0-85059497244, 68. 30609658 PMC6357185

[37] Irby D. , Du C. , and Li F. , Lipid–Drug Conjugate for Enhancing Drug Delivery, Molecular Pharmaceutics. (2017) 14, no. 5, 1325–1338, 10.1021/acs.molpharmaceut.6b01027, 2-s2.0-85018445598. 28080053 PMC5477224

[38] Kaushik D. , Verma R. , Rao R. , and Kaushik P. , Introduction to Lipid-Based Drug Delivery Systems for Oral Delivery, A Comprehensive Text Book on Self-Emulsifying Drug Delivery Systems, 2021, Bentham Science Publishers, 30–58.

[39] Lazăr A. R. , Pușcaș A. , Tanislav A. E. , and Mureșan V. , Bioactive Compounds Delivery and Bioavailability in Structured Edible Oils Systems, Comprehensive Reviews in Food Science and Food Safety. (2024) 23, no. 6, 10.1111/1541-4337.70020, e70020. 39437192

[40] Esen R. , Öz M. , and Dikel S. , Effects of Artichoke (*Cynara scolymus*) Leaf Extract on the Growth, Blood, and Biochemistry Parameters of Nile Tilapia (*Oreochromis niloticus*), Tropical Animal Health and Production. (2025) 57, no. 6, 10.1007/s11250-025-04536-y, 284. 40555873 PMC12187807

[41] Öz M. , Inanan B. E. , Üstüner E. , Karagoz B. , and Dikel S. , Effects of Dietary Garlic (*Allium sativum*) Oil on Growth Performance, Haemato-Biochemical and Histopathology of Cypermethrin-Intoxicated Nile Tilapia (*Oreochromis niloticus*), Veterinary Medicine and Science. (2024) 10, no. 3, 10.1002/vms3.1449, e1449. 38581350 PMC10998455

[42] Öz M. , Üstüner E. , and Bölükbaş F. , Effects of Dietary Black Cumin (*Nigella sativa* L.) Oil on Growth Performance, Hemato-Biochemical and Histopathology of Cypermethrin-Intoxicated Nile Tilapia (*Oreochromis niloticus*), Journal of the World Aquaculture Society. (2024) 55, no. 1, 273–288, 10.1111/jwas.13005.

[43] Öz M. , Üstüner E. , Jumayeva M. , and Dikel S. , Dietary *Nigella sativa* Oil Confers Protection Against Diazinon Toxicity in Nile Tilapia (*Oreochromis niloticus*): A Detoxification-Based Approach, Veterinary Research Communications. (2025) 49, no. 5, 10.1007/s11259-025-10861-2, 305. 40924273

[44] Tok S. , Öz M. , and Dikel S. , Effects of Dietary Anise (*Pimpinella anisum* L.) Oil on Growth Performance, Blood Parameters and Muscle Nutrient Content of Nile Tilapia (*Oreochromis niloticus*), Veterinary Medicine and Science. (2025) 11, no. 4, 10.1002/vms3.70443, e70443. 40454886 PMC12128472

[45] Dawood M. A. , Basuini M. F. El , and Yilmaz S. , et al.Exploring the Roles of Dietary Herbal Essential Oils in Aquaculture: A Review, Animals. (2022) 12, no. 7, 10.3390/ani12070823, 823. 35405814 PMC8996993

[46] Mbokane E. M. and Moyo N. A. G. , Use of Medicinal Plants as Feed Additives in the Diets of Mozambique Tilapia (*Oreochromis mossambicus*) and the African Sharptooth Catfish (*Clarias gariepinus*) in Southern Africa, Frontiers in Veterinary Science. (2022) 9, 10.3389/fvets.2022.1072369, 1072369. 36590800 PMC9800439

[47] Miljanović A. , Grbin D. , and Pavić D. , et al.Essential Oils of Sage, Rosemary, and Bay Laurel Inhibit the Life Stages of Oomycete Pathogens Important in Aquaculture, Plants. (2021) 10, no. 8, 10.3390/plants10081676, 1676. 34451721 PMC8401702

[48] Valdivieso-Ugarte M. , Gomez-Llorente C. , Plaza-Díaz J. , and Gil Á. , Antimicrobial, Antioxidant, and Immunomodulatory Properties of Essential Oils: A Systematic Review, Nutrients. (2019) 11, no. 11, 10.3390/nu11112786, 2786. 31731683 PMC6893664

[49] Ghamkhar R. and Hicks A. , Sustainable Aquafeeds: Using Aquafarmer Preference to Inform a Multi-criteria Decision Analysis, ACS Agricultural Science & Technology. (2021) 1, no. 3, 270–280, 10.1021/acsagscitech.1c00053.

[50] Valentim D. , Duarte J. , and Oliveira A. , et al.Nanoemulsion From Essential Oil of *Pterodon emarginatus* (Fabaceae) Shows In Vitro Efficacy Against Monogeneans of *Colossoma macropomum* (Pisces: Serrasalmidae), Journal of Fish Diseases. (2018) 41, no. 3, 443–449, 10.1111/jfd.12739, 2-s2.0-85036549767. 29194663

[51] Abdel-Latif H. M. R. , Abdel-Tawwab M. , Khafaga A. F. , and Dawood M. A. O. , Dietary Origanum Essential Oil Improved Antioxidative Status, Immune-Related Genes, and Resistance of Common Carp (*Cyprinus carpio* L.) to *Aeromonas hydrophila* Infection, Fish & Shellfish Immunology. (2020) 104, 1–7, 10.1016/j.fsi.2020.05.056. 32474085

[52] Alagawany M. , Farag M. R. , Salah A. S. , and Mahmoud M. A. , The Role of Oregano Herb and Its Derivatives as Immunomodulators in Fish, Reviews in Aquaculture. (2020) 12, no. 4, 2481–2492, 10.1111/raq.12453.

[53] Chbel A. , Elmakssoudi A. , Rey-Méndez M. , Barja J. L. , Soukri A. , and El khalfi B. , Analysis of the Chemical Compositions of Six Essential Oils and Evaluation of Their Antioxidant and Antibacterial Activities Against Some Drug-Resistant Bacteria in Aquaculture, Journal of Herbmed Pharmacology. (2022) 11, no. 3, 401–408, 10.34172/jhp.2022.46.

[54] Diler O. , Gormez O. , Diler I. , and Metin S. , Effect of Oregano (*Origanum onites* L.) Essential Oil on Growth, Lysozyme and Antioxidant Activity and Resistance Against *Lactococcus garvieae* in Rainbow Trout, *Oncorhynchus mykiss* (Walbaum), Aquaculture Nutrition. (2017) 23, no. 4, 844–851, 10.1111/anu.12451, 2-s2.0-84994525489.

[55] Ghafarifarsani H. , Hoseinifar S. H. , and Sheikhlar A. , et al.The Effects of Dietary Thyme Oil (*Thymus vulgaris*) Essential Oils for Common Carp (*Cyprinus carpio*): Growth Performance, Digestive Enzyme Activity, Antioxidant Defense, Tissue and Mucus Immune Parameters, and Resistance Against *Aeromonas hydrophila* , Aquaculture Nutrition. (2022) 2022, no. 1, 10.1155/2022/7942506, 7942506. 36860465 PMC9973159

[56] Magouz F. I. , Shehab El-Din M. T. , Amer A. A. , Gewaily M. S. , El-Dahdoh W. A. , and Dawood M. A. O. , A Blend of Herbal Essential Oils Enhanced the Growth Performance, Blood Bio-Immunology Traits, and Intestinal Health of Nile Tilapia (*Oreochromis niloticus*), Annals of Animal Science. (2022) 22, no. 2, 751–761, 10.2478/aoas-2021-0066.

[57] Mandalakis M. , Anastasiou T. I. , and Martou N. , et al.Antibacterial Effects of Essential Oils of Seven Medicinal-Aromatic Plants Against the Fish Pathogen *Aeromonas veronii* Bv. Sobria: To Blend or Not to Blend?, Molecules. (2021) 26, no. 9, 2731. 34066575 10.3390/molecules26092731 PMC8125735

[58] Moustafa E. M. , Shukry M. , and Assas M. , et al.Dietary Oregano Essential Oil and Sodium Butyrate Enhance Growth, Immunity, and Gene Expression in Nile Tilapia Post-*Aeromonas hydrophila* Infection, Scientific Reports. (2025) 15, no. 1, 10.1038/s41598-025-22439-8, 38496. 41188425 PMC12586456

[59] Syanya F. J. , Winam Z. O. , Khanna A. N. , Mahadevan H. , Lovejan M. , and Mumina P. , Effects of Dietary Oregano (*Origanum vulgare*) Essential Oil Supplementation in Hybrid Red Tilapia (Oreochromis Spp.) Diet on Growth Performance, Survival, Intestinal Health, Reproductive and Serum Biochemical Indices, Aquaculture Research. (2025) 2025, no. 1, 4938650.

[60] Anastasiou T. I. , Mandalakis M. , and Krigas N. , et al.Comparative Evaluation of Essential Oils From Medicinal-Aromatic Plants of Greece: Chemical Composition, Antioxidant Capacity and Antimicrobial Activity Against Bacterial Fish Pathogens, Molecules. (2019) 25, no. 1, 10.3390/molecules25010148, 148. 31905915 PMC6982863

[61] Ghafarifarsani H. , Aftabgard M. , Hoseinifar S. H. , Raeeszadeh M. , and Van Doan H. , Comparative Effects of Savory (*Satureja hortensis*), Dill (*Anethum graveolens*), and Mooseer (Allium Hirtifolium) Essential Oils on Growth, Digestive, and Immunoantioxidant Parameters and Resistance to *Aeromonas hydrophila* in Juvenile Common Carp (*Cyprinus carpio*), Aquaculture. (2023) 572, 739541.

[62] Jasim S. A. , Davoodi R. , and Yasin G. , et al.Potential Protective Effects of Thyme (*Thymus vulgaris*) Essential Oil on Growth, Hematology, Immune Responses, and Antioxidant Status of *Oncorhynchus mykiss* Exposed to Malathion, Annals of Animal Science. (2023) 23, no. 2, 481–493, 10.2478/aoas-2022-0064.

[63] Terrazas-Pineda K. A. , Alamilla-Beltrán L. , and Acero-Ortega C. A. , et al.Antimicrobial Activity of Cinnamon, Tea Tree, and Thyme Essential Oils Against Pathogenic Bacteria Isolated From Tilapia (*Oreochromis* spp.) in Aquaculture Farms, Molecules. (2025) 30, no. 13, 10.3390/molecules30132799, 2799. 40649314 PMC12250716

[64] Snuossi M. , Trabelsi N. , Ben Taleb S. , Dehmeni A. , Flamini G. , and De Feo V. , *Laurus nobilis*, *Zingiber officinale* and *Anethum graveolens* Essential Oils: Composition, Antioxidant and Antibacterial Activities Against Bacteria Isolated From Fish and Shellfish, Molecules. (2016) 21, no. 10, 10.3390/molecules21101414, 2-s2.0-84994009742, 1414. 27782086 PMC6273486

[65] Al-Sagheer A. A. , Mahmoud H. K. , Reda F. M. , Mahgoub S. A. , and Ayyat M. S. , Supplementation of Diets for *Oreochromis niloticus* With Essential Oil Extracts From Lemongrass (*Cymbopogon citratus*) and Geranium (*Pelargonium graveolens*) and Effects on Growth, Intestinal Microbiota, Antioxidant and Immune Activities, Aquaculture Nutrition. (2018) 24, no. 3, 1006–1014, 10.1111/anu.12637, 2-s2.0-85031398314.

[66] de Oliveira I. C. , Oliveira R. S. M. , and da Paixão Lemos C. H. , et al.Essential Oils From *Cymbopogon citratus* and Lippia Sidoides in the Anesthetic Induction and Transport of Ornamental Fish *Pterophyllum scalare* , Fish Physiology and Biochemistry. (2022) 48, no. 3, 501–519, 10.1007/s10695-022-01075-3. 35435543

[67] Aydın B. and Barbas L. A. L. , Sedative and Anesthetic Properties of Essential Oils and Their Active Compounds in Fish: A Review, Aquaculture. (2020) 520, 10.1016/j.aquaculture.2020.734999, 734999.

[68] de Souza Silva L. T. , de Pádua Pereira U. , and de Oliveira H. M. , et al.Hemato-Immunological and Zootechnical Parameters of Nile Tilapia Fed Essential Oil of *Mentha piperita* After Challenge With *Streptococcus agalactiae* , Aquaculture. (2019) 506, 205–211, 10.1016/j.aquaculture.2019.03.035, 2-s2.0-85063328626.

[69] Magouz F. I. , Mahmoud S. A. , and El-Morsy R. A. , et al.Dietary Menthol Essential Oil Enhanced the Growth Performance, Digestive Enzyme Activity, Immune-Related Genes, and Resistance Against Acute Ammonia Exposure in Nile Tilapia (*Oreochromis niloticus*), Aquaculture. (2021) 530, 10.1016/j.aquaculture.2020.735944, 735944.

[70] Valladao G. M. , Gallani S. U. , and Pala G. , et al.Practical Diets With Essential Oils of Plants Activate the Complement System and Alter the Intestinal Morphology of Nile Tilapia, Aquaculture Research. (2017) 48, no. 11, 5640–5649, 10.1111/are.13386, 2-s2.0-85019933500.

[71] Srinivasan R. , Jin X. , Lin X. , and Zhao Z. , Essential Oil Compounds as Antibiotic Alternatives: A Comprehensive Review of Antibacterial, Anti-Quorum Sensing, and Antibiofilm Effects Against *Vibrio* spp. in Aquaculture, Reviews in Aquaculture. (2025) 17, no. 4, 10.1111/raq.70065, e70065.

[72] Das R. , Thaosen N. , Sarma K. , and Sarma D. , Essential Oil of *Curcuma aromatica* as a Dietary Supplement: Evaluation of Its Effect on Growth, Haematology, Immune Response and Histopathology in *Channa punctata* Challenged With Two Pathogenic Aeromonads, Animal Feed Science and Technology. (2025) 321, 10.1016/j.anifeedsci.2025.116235, 116235.

[73] Vanderzwalmen M. , Eaton L. , and Mullen C. , et al.The use of Feed and Water Additives for Live Fish Transport, Reviews in Aquaculture. (2019) 11, no. 1, 263–278, 10.1111/raq.12239, 2-s2.0-85061506339.

[74] Acar Ü. , Yıldırım Ö. , Baba E. , Navruz F. Z. , Hacisa M. , and Yılmaz S. , Effects of a Myrrh (Commiphora myrrha) Essential Oil Supplemented Diet on Haemato-biochemical Parameters, Expression of Tissue-Specific Immune-and Stress-related Genes, And Resistance of Cyprinus Carpio to Aeromonas Hydrophila Infection, Turkish Journal of Fisheries and Aquatic Sciences. (2025) 25, no. 8, 10.4194/TRJFAS27525.

[75] Hajirezaee S. and Khanjani M. H. , Evaluation of Dietary Inclusion of *Bunium persicum*, *Bunium persicum* Essential Oil on Growth, Immune Components, Immune-Related Gene Expressions and Resistance to *Aeromonas hydrophila*, in Rainbow Trout, *Oncorhynchus mykiss* , Aquaculture Research. (2021) 52, no. 10, 4711–4723, 10.1111/are.15305.

[76] Hajirezaee S. , Rohanizadehghadikolaei F. , Afzali-Kordmahalleh A. , and Khanjani M. H. , Effects of Dietary Common Juniper (*Juniperus communis*) Essential Oil on Growth, Immunity, Antioxidant Status, and Disease Resistance in Rainbow Trout, *Oncorhynchus mykiss* , Aquaculture Reports. (2024) 34, 10.1016/j.aqrep.2023.101895, 101895.

[77] Miura P. T. , Queiroz S. C. N. , Jonsson C. M. , Chagas E. C. , Chaves F. C. M. , and Reyes F. G. , Study of the Chemical Composition and Ecotoxicological Evaluation of Essential Oils in *Daphnia magna* with Potential use in Aquaculture, Aquaculture Research. (2021) 52, no. 7, 3415–3424, 10.1111/are.15186.

[78] Abdo S. E. , El-Nahas A. F. , and Abdellatif R. E. , et al.Combined Dietary Spirulina Platensis and *Citrus limon* Essential Oil Enhances the Growth, Immunity, Antioxidant Capacity and Intestinal Health of Nile Tilapia, Veterinary Sciences. (2024) 11, no. 10, 10.3390/vetsci11100474, 474. 39453066 PMC11512375

[79] Acar Ü. , Kesbiç O. S. , Yılmaz S. , Gültepe N. , and Türker A. , Evaluation of the Effects of Essential Oil Extracted From Sweet Orange Peel (*Citrus sinensis*) on Growth Rate of Tilapia (*Oreochromis mossambicus*) and Possible Disease Resistance against *Streptococcus iniae* , Aquaculture. (2015) 437, 282–286, 10.1016/j.aquaculture.2014.12.015, 2-s2.0-84919935353.

[80] Baba E. , Acar Ü. , Öntaş C. , Kesbiç O. S. , and Yılmaz S. , Evaluation of *Citrus limon* Peels Essential Oil on Growth Performance, Immune Response of Mozambique Tilapia *Oreochromis mossambicus* Challenged With *Edwardsiella tarda* , Aquaculture. (2016) 465, 13–18, 10.1016/j.aquaculture.2016.08.023, 2-s2.0-84983405693.

[81] d. A. Pereira Júnior J. , Costa D. S. , and d. S. d. Silva A. , et al.Enriched Diet With Orange Essential Oil *Citrus sinensis* for Tambaqui *Colossoma macropomum* Promotes Growth Performance and Resistance Against *Aeromonas hydrophila* , Journal of Fish Diseases. (2025) 48, no. 1, 10.1111/jfd.14039, e14039. 39470138

[82] Ham Y. , Yang J. , Choi W.-S. , Ahn B.-J. , and Park M.-J. , Antibacterial Activity of Essential Oils From Pinaceae Leaves Against Fish Pathogens, Journal of the Korean Wood Science and Technology. (2020) 48, no. 4, 527–547, 10.5658/WOOD.2020.48.4.527.

[83] Mathew R. T. , Alkhamis Y. A. , and Alngada R. S. , et al.Dose Response Effects of Dietary Clove and Peppermint Oils on the Growth Performance, Physio-Metabolic Response, Feed Utilization, Immunity, and Organ Histology in African Catfish (*clarias gariepinus*), Veterinary Research Communications. (2025) 49, no. 2, 10.1007/s11259-025-10660-9, 101. 39920512

[84] Ning L. , Zhang X. , Zhang D. , Hu Y. , and Li Y. , The Benefits of Blend Essential Oil for GIFT Tilapia on the Digestion, Antioxidant, and Muscle Quality During Cold Storage, Aquaculture. (2021) 533, 10.1016/j.aquaculture.2020.736097, 736097.

[85] Kaushik D. , Verma R. , Rao R. , and Kaushik P. , Introduction to Lipid-Based Drug Delivery Systems for Oral Delivery, A Comprehensive Text Book on Self-emulsifying Drug Delivery Systems, 2021, 30.

[86] Nahum V. and Domb A. J. , Recent Developments in Solid Lipid Microparticles for Food Ingredients Delivery, Foods. (2021) 10, no. 2, 10.3390/foods10020400, 400. 33670356 PMC7917609

[87] McClements D. J. and Öztürk B. , Utilization of Nanotechnology to Improve the Handling, Storage and Biocompatibility of Bioactive Lipids in Food Applications, Foods. (2021) 10, no. 2, 10.3390/foods10020365, 365. 33567622 PMC7915003

[88] Yang J. and Ciftci O. N. , Encapsulation of Fish Oil Into Hollow Solid Lipid Micro-and Nanoparticles Using Carbon Dioxide, Food Chemistry. (2017) 231, 105–113, 10.1016/j.foodchem.2017.03.109, 2-s2.0-85016241939. 28449985

[89] Vaseeharan B. and Thaya R. , Medicinal Plant Derivatives as Immunostimulants: An Alternative to Chemotherapeutics and Antibiotics in Aquaculture, Aquaculture International. (2014) 22, no. 3, 1079–1091, 10.1007/s10499-013-9729-3, 2-s2.0-84900034708.

[90] Liang J. , Zhang Y. , and Chi P. , et al.Essential Oils: Chemical Constituents, Potential Neuropharmacological Effects and Aromatherapy - A review, Pharmacological Research - Modern Chinese Medicine. (2023) 6, 10.1016/j.prmcm.2022.100210, 100210.

[91] Nguyen N. P. K. , Tran K. N. , Nguyen L. T. H. , Shin H.-M. , and Yang I.-J. , Effects of Essential Oils and Fragrant Compounds on Appetite: A Systematic Review, International Journal of Molecular Sciences. (2023) 24, no. 9, 10.3390/ijms24097962, 7962. 37175666 PMC10178777

[92] Liu C. , Zhao L.-P. , and Shen Y.-Q. , A Systematic Review of Advances in Intestinal Microflora of Fish, Fish Physiology and Biochemistry. (2021) 47, no. 6, 2041–2053, 10.1007/s10695-021-01027-3. 34750711

[93] Hoseinifar S. H. , Van Doan H. , Dadar M. , Ringø E. , and Harikrishnan R. , Feed Additives, Gut Microbiota, and Health in Finfish Aquaculture, Microbial Communities in Aquaculture Ecosystems: Improving Productivity and Sustainability, 2019, Springer, 121–142.

[94] Zaminhan-Hassemer M. , Zagolin G. B. , and Perazza C. A. , et al.Adding an Essential Oil Blend to the Diet of Juvenile Nile Tilapia Improves Growth and Alters the Gut Microbiota, Aquaculture. (2022) 560, 10.1016/j.aquaculture.2022.738581, 738581.

[95] Butt R. L. and Volkoff H. , Gut Microbiota and Energy Homeostasis in Fish, Frontiers in Endocrinology. (2019) 10, 10.3389/fendo.2019.00009, 2-s2.0-85064223984, 9. 30733706 PMC6353785

[96] Agustí A. , García-Pardo M. P. , and López-Almela I. , et al.Interplay Between the Gut-Brain Axis, Obesity and Cognitive Function, Frontiers in Neuroscience. (2018) 12, 10.3389/fnins.2018.00155, 2-s2.0-85044054473, 329519. PMC5864897 29615850

[97] Öz M. , Inanan B. E. , Karasahin T. , and Dikel S. , Effects of Glutamine on Growth Performance, Nutrient Content, Fatty Acid Profile, and Blood Parameters of Rainbow Trout (*Oncorhynchus mykiss*), Journal of Fish Biology. (2024) 104, no. 4, 1213–1222, 10.1111/jfb.15666. 38263635

[98] Reindl K. M. and Sheridan M. A. , Peripheral Regulation of the Growth Hormone-Insulin-Like Growth Factor System in Fish and Other Vertebrates, Comparative Biochemistry and Physiology Part A: Molecular & Integrative Physiology. (2012) 163, no. 3-4, 231–245, 10.1016/j.cbpa.2012.08.003, 2-s2.0-84866357104. 22909791

[99] Aanyu M. , Betancor M. B. , and Monroig O. , Effects of Dietary Limonene and Thymol on the Growth and Nutritional Physiology of Nile Tilapia (*Oreochromis niloticus*), Aquaculture. (2018) 488, 217–226, 10.1016/j.aquaculture.2018.01.036, 2-s2.0-85041659421.

[100] Midhun S. J. , Arun D. , and Edatt L. , et al.Modulation of Digestive Enzymes, GH, IGF-1 and IGF-2 Genes in the Teleost, Tilapia (*Oreochromis mossambicus*) by Dietary Curcumin, Aquaculture International. (2016) 24, no. 5, 1277–1286, 10.1007/s10499-016-9984-1, 2-s2.0-84959342587.

[101] Tian P. , Huo L. , and Shi Q. , et al.Essential Oils Promote the Growth Performance of Grass Carp, Chinese Soft-Shelled Turtles, and Zebrafish, Aquaculture International. (2025) 33, no. 1, 10.1007/s10499-024-01699-7, 59.

[102] Fadeifard F. , Raissy M. , Jafarian M. , Boroujeni H. R. , Rahimi M. , and Faghani M. , Effects of Black Seed (*Nigella sativa*), Ginger (*Zingiber officinale*) and Cone Flower (*Echinacea angustifolia*) on the Immune System of Rainbow Trout, *Oncorhynchus mykiss* , Arquivo Brasileiro de Medicina Veterinária e Zootecnia. (2018) 70, no. 1, 199–204, 10.1590/1678-4162-8489, 2-s2.0-85046018693.

[103] d. F. Souza C. , Baldissera M. D. , Baldisserotto B. , Heinzmann B. M. , Martos-Sitcha J. A. , and Mancera J. M. , Essential Oils as Stress-Reducing Agents for Fish Aquaculture: A Review, Frontiers in Physiology. (2019) 10, 10.3389/fphys.2019.00785, 2-s2.0-85069788799, 785. 31281264 PMC6596282

[104] Kelly C. and Salinas I. , Under Pressure: Interactions Between Commensal Microbiota and the Teleost Immune System, Frontiers in Immunology. (2017) 8, 10.3389/fimmu.2017.00559, 2-s2.0-85019974336, 559. 28555138 PMC5430139

[105] da Silva E. , Deschamps G. T. , and d. L. Matter F. , et al.The Anaesthetic Efficacy of *Eucalyptus globulus* Essential Oil on Silver Catfish (*Rhamdia quelen*), Aquaculture Research. (2021) 52, no. 11, 5190–5197, 10.1111/are.15388.

[106] Taheri Mirghaed A. , Hoseini S. M. , Aydın B. , Paolucci M. , Hoseinifar S. H. , and Van Doan H. , Effects of Anaesthesia With 1, 8-Cineole on Haematological and Plasma Stress Responses in Caspian Trout, *Salmo caspius*, Subadults, Aquaculture Research. (2022) 53, no. 3, 893–900, 10.1111/are.15631.

[107] Fu Y. W. , Yao Z. J. , and He M. H. , et al.Expression Analysis and Tissue Localization of IgZ in the Grouper *Epinephelus coioides* After Vibrio Alginolyticus Infection and Vaccination, Journal of Fish Diseases. (2021) 44, no. 10, 1647–1655, 10.1111/jfd.13471. 34133777

[108] Mai T. T. , Kayansamruaj P. , and Taengphu S. , et al.Efficacy of Heat-Killed and Formalin-Killed Vaccines Against Tilapia Tilapinevirus in Juvenile Nile Tilapia (*Oreochromis niloticus*), Journal of Fish Diseases. (2021) 44, no. 12, 2097–2109, 10.1111/jfd.13523. 34477227 PMC9291230

[109] Matz H. , Taylor R. S. , and Redmond A. K. , et al.Organized B Cell Sites in Cartilaginous Fishes Reveal the Evolutionary Foundation of Germinal Centers, Cell Reports. (2023) 42, no. 7, 10.1016/j.celrep.2023.112664, 112664. 37342909 PMC10529500

[110] Shibasaki Y. , Afanasyev S. , and Fernández-Montero A. , et al.Cold-Blooded Vertebrates Evolved Organized Germinal Center–like Structures, Science Immunology. (2023) 8, no. 90, 10.1126/sciimmunol.adf1627, eadf1627. 37910630 PMC11152321

[111] Meira C. M. , Carriero M. M. , and Pereira N. L. , et al.Immunological Effects of DNA Vaccination and Interleukin Utilization as an Adjuvant in *Astyanax lacustris* Immunized Against *Ichthyophthirius multifiliis* , Journal of Fish Diseases. (2024) 47, no. 9, 10.1111/jfd.13979, e13979. 38879867

[112] Peixoto D. , Carvalho I. , and Cunha A. , et al.Synergistic Effects of Dietary Tryptophan and Dip Vaccination in the Immune Response of European Seabass Juveniles, International Journal of Molecular Sciences. (2024) 25, no. 22, 10.3390/ijms252212200, 12200. 39596266 PMC11595104

[113] Sayed M. , Essa M. , and Abdelhamed H. , Persistence and Immunogenicity of Edwardsiella Piscicida phoP/Q Mutants in Channel Catfish (*Ictalurus punctatus*), Journal of Veterinary Medical Research. (2022) 29, no. 2, 43–51, 10.21608/jvmr.2022.135472.1057.

[114] Wang E. , Qin Z. , and Yu Z. , et al.Molecular Characterization, Phylogenetic, Expression, and Protective Immunity Analysis of OmpF, a Promising Candidate Immunogen Against *Yersinia ruckeri* Infection in Channel Catfish, Frontiers in Immunology. (2018) 9, 10.3389/fimmu.2018.02003, 2-s2.0-85054356201, 2003. 30271401 PMC6146100

[115] Wei Q. , Li H. , and Chen Y. , et al.The Pilot Study on the Histological and Ultrastructural Characteristics of Major Immune Organs (Spleen, Head Kidney, and Trunk Kidney) and Analysis of Pathological Features in Sichuan Taimen (*Hucho bleekeri*), Journal of Fish Diseases. (2025) 48, no. 12, 10.1111/jfd.14168, e14168. 40485491

[116] Samaï H. C. , Rioult D. , and Bado-Nilles A. , et al.Procedures for Leukocytes Isolation From Lymphoid Tissues and Consequences on Immune Endpoints Used to Evaluate Fish Immune Status: A Case Study on Roach (*Rutilus rutilus*), Fish & Shellfish Immunology. (2018) 74, 190–204, 10.1016/j.fsi.2017.12.040, 2-s2.0-85042704477. 29288813

[117] Firmino J. P. , Fernández-Alacid L. , and Vallejos-Vidal E. , et al.Carvacrol, Thymol, and Garlic Essential Oil Promote Skin Innate Immunity in Gilthead Seabream (*Sparus aurata*) Through the Multifactorial Modulation of the Secretory Pathway and Enhancement of Mucus Protective Capacity, Frontiers in Immunology. (2021) 12, 10.3389/fimmu.2021.633621, 633621. 33777020 PMC7994269

[118] Hajirezaee S. , Khanjani M. H. , Ahani S. , and Ghiasvand Z. , Tarragon (*Artemisia dracunculus*) Essential Oil at Optimized Dietary Levels Prompted Growth, Immunity, and Resistance to Enteric Red-Mouth Disease in the Rainbow Trout (*Oncorhynchus mykiss*), Aquaculture Research. (2024) 2024, no. 1, 10.1155/2024/3273850, 3273850.

[119] Campoverde C. , Milne D. J. , Estévez A. , Duncan N. , Secombes C. J. , and Andree K. B. , Ontogeny and Modulation After PAMPs Stimulation of β-Defensin, Hepcidin, and Piscidin Antimicrobial Peptides in Meagre (*Argyrosomus regius*), Fish & Shellfish Immunology. (2017) 69, 200–210, 10.1016/j.fsi.2017.08.026, 2-s2.0-85028070384. 28842373

[120] Ordóñez-Grande B. , Fernández-Alacid L. , and Sanahuja I. , et al.Evaluating Mucus Exudation Dynamics Through Isotopic Enrichment and Turnover of Skin Mucus Fractions in a Marine Fish Model, Conservation Physiology. (2020) 8, no. 1, 10.1093/conphys/coaa095, coaa095. 33442471 PMC7787050

[121] Öz M. , Üstüner E. , and Dikel S. , The Protective Role of Dietary Black Cumin (*Nigella sativa* L.) Oil Against Waterborne Boric Acid in Nile Tilapia (*Oreochromis niloticus*): Hematological, Biochemical, Oxidative Stress, and Histopathological Responses, Aquaculture International. (2026) 34, no. 1, 10.1007/s10499-025-02422-w, 19.

[122] Andrés C. M. C. , de la Lastra J. M. P. , Munguira E. B. , Juan C. A. , Plou F. J. , and Lebeña E. P. , Electrophilic Compounds in the Human Diet and Their Role in the Induction of the Transcription Factor NRF2, International Journal of Molecular Sciences. (2024) 25, no. 6, 10.3390/ijms25063521, 3521. 38542492 PMC10971185

[123] Fuse Y. and Kobayashi M. , Conservation of the Keap1-Nrf2 System: An Evolutionary Journey Through Stressful Space and Time, Molecules. (2017) 22, no. 3, 10.3390/molecules22030436, 2-s2.0-85015719341, 436. 28282941 PMC6155405

[124] Wang M. , He Z. , Xiong Z. , Liu H. , Zhou X. , and He J. , “Dietary Grape Seed Extract, Onion Peel Extract or Rosemary Extract Supplementation Alleviates Diquat-Induced Restrained Growth and Oxidative Stress of Lohmann Chicks, 2022. 10.1080/10495398.2023.2271532 37878368

[125] Yang Z. , Sun G. , and Tao J. , et al.Dietary Tea Polyphenols Improve Growth Performance and Intestinal Microbiota Under Chronic Crowding Stress in Hybrid Crucian Carp, Animals. (2025) 15, no. 13, 10.3390/ani15131983, 1983. 40646882 PMC12248730

[126] Cheng K. , Huang Y. , and Wang C. , 1,25(OH)2D3 Inhibited Ferroptosis in Zebrafish Liver Cells (ZFL) by Regulating Keap1-Nrf2-GPx4 and NF-κB-hepcidin Axis, International Journal of Molecular Sciences. (2021) 22, no. 21, 10.3390/ijms222111334, 11334. 34768761 PMC8583391

[127] Gugliandolo A. , Bramanti P. , and Mazzon E. , Activation of Nrf2 by Natural Bioactive Compounds: A Promising Approach for Stroke?, International Journal of Molecular Sciences. (2020) 21, no. 14, 10.3390/ijms21144875, 4875. 32664226 PMC7402299

[128] Ibrahim D. , Abd El-Hamid M. I. , and Al-Zaban M. I. , et al.Impacts of Fortifying Nile Tilapia (*Oreochromis niloticus*) Diet With Different Strains of Microalgae on Its Performance, Fillet Quality and Disease Resistance to *Aeromonas hydrophila* considering the Interplay Between Antioxidant and Inflammatory Response, Antioxidants. (2022) 11, no. 11, 10.3390/antiox11112181, 2181. 36358553 PMC9686914

[129] Torrecillas S. , Terova G. , and Makol A. , et al.Dietary Phytogenics and Galactomannan Oligosaccharides in Low Fish Meal and Fish Oil-Based Diets for European Sea Bass (*Dicentrarchus labrax*) Juveniles: Effects on Gill Structure and Health and Implications on Oxidative Stress Status, Frontiers in Immunology. (2021) 12, 10.3389/fimmu.2021.663106, 663106. 34054829 PMC8149968

[130] Özel O. , Düzgüneş Z. , Gürkan S. , and Çakmak E. , Impact of Oregano (*Origanum vulgare*) Supplementation on Antioxidant Status, and Related Gene Expression in Black Sea Salmon, *Salmo labrax* , Journal of the Hellenic Veterinary Medical Society. (2024) 75, no. 2, 7217–7226, 10.12681/jhvms.31526.

[131] Singh M. K. , Borah D. , and Dutta M. P. , et al.A Review on Immunostimulatory and Antioxidant Potential of Herbs, *Curcuma longa* L., *Camellia sinensis* L. *Zingiber officinale* and *Allium sativum* Linn. in Fish Health: A Sustainable Approach for a Healthy Aquaculture, Ecology, Environment and Conservation. (2022) 28, no. 3, 1431–1445, 10.53550/EEC.2022.v28i03.047.

[132] Zahran E. , Elbahnaswy S. , Ahmed F. , Ibrahim I. , Khaled A. A. , and Eldessouki E. A. , Nutritional and Immunological Evaluation of Nannochloropsis Oculata as a Potential Nile Tilapia-Aquafeed Supplement, BMC Veterinary Research. (2023) 19, no. 1, 10.1186/s12917-023-03618-z, 65. 37076908 PMC10114411

[133] Wu F. , Huang W. , and Liu Q. , et al.Responses of Antioxidant Defense and Immune Gene Expression in Early Life Stages of Large Yellow Croaker (Pseudosciaena Crocea) Under Methyl Mercury Exposure, Frontiers in Physiology. (2018) 9, 10.3389/fphys.2018.01436, 2-s2.0-85055089474, 1436. 30364149 PMC6191496

[134] Fu X. , Li K. , and Niu Y. , et al.The mTOR/PGC-1α/SIRT3 Pathway Drives Reductive Glutamine Metabolism to Reduce Oxidative Stress Caused by ISKNV in CPB Cells, Microbiology Spectrum. (2022) 10, no. 1, e02310–e02321, 10.1128/spectrum.02310-21. 35019690 PMC8754121

[135] Peng J. , Yang Z. , and Li H. , et al.Quercetin Reprograms Immunometabolism of Macrophages via the SIRT1/PGC-1α Signaling Pathway to Ameliorate Lipopolysaccharide-Induced Oxidative Damage, International Journal of Molecular Sciences. (2023) 24, no. 6, 10.3390/ijms24065542, 5542. 36982615 PMC10059595

[136] Kheawfu K. , Pikulkaew S. , and Wellendorph P. , et al.Elucidating Pathway and Anesthetic Mechanism of Action of Clove Oil Nanoformulations in Fish, Pharmaceutics. (2022) 14, no. 5, 10.3390/pharmaceutics14050919, 919. 35631505 PMC9147060

[137] Mansour A. T. , Mahboub H. H. , and Elshopakey G. E. , et al.Physiological Performance, Antioxidant and Immune Status, Columnaris Resistance, and Growth of Nile Tilapia that Received *Alchemilla vulgaris*-Supplemented Diets, Antioxidants. (2022) 11, no. 8, 10.3390/antiox11081494, 1494. 36009213 PMC9404728

[138] Tang Y. , Zhang H. , and Yang G. , et al.Pharmacokinetics Studies of Eugenol in Pacific White Shrimp (*Litopenaeus vannamei*) After Immersion Bath, BMC Veterinary Research. (2022) 18, no. 1, 10.1186/s12917-022-03145-3, 122. 35361203 PMC8969250

[139] Cantas L. , Sørby J. R. T. , Aleström P. , and Sørum H. , Culturable Gut Microbiota Diversity in Zebrafish, Zebrafish. (2012) 9, no. 1, 26–37, 10.1089/zeb.2011.0712, 2-s2.0-84859060167. 22428747 PMC3308716

[140] de Freitas Souza C. , Baldissera M. D. , and Bianchini A. E. , et al.Citral and Linalool Chemotypes of *Lippia alba* Essential Oil as Anesthetics for Fish: A Detailed Physiological Analysis of Side Effects During Anesthetic Recovery in Silver Catfish (*Rhamdia quelen*), Fish Physiology and Biochemistry. (2018) 44, no. 1, 21–34, 10.1007/s10695-017-0410-z, 2-s2.0-85029817189. 28948452

[141] Hoseini S. M. , Taheri Mirghaed A. , and Yousefi M. , Application of Herbal Anaesthetics in Aquaculture, Reviews in Aquaculture. (2019) 11, no. 3, 550–564, 10.1111/raq.12245, 2-s2.0-85046652546.

[142] Dawood M. A. , El Basuini M. F. , and Zaineldin A. I. , et al.Antiparasitic and Antibacterial Functionality of Essential Oils: An Alternative Approach for Sustainable Aquaculture, Pathogens. (2021) 10, no. 2, 10.3390/pathogens10020185, 185. 33572193 PMC7914417

[143] Domínguez-Borbor C. , Sánchez-Rodríguez A. , Sonnenholzner S. , and Rodríguez J. , Essential Oils Mediated Antivirulence Therapy Against Vibriosis in Penaeus Vannamei, Aquaculture. (2020) 529, 10.1016/j.aquaculture.2020.735639, 735639.

[144] Gholipourkanani H. , Buller N. , and Lymbery A. , In Vitro Antibacterial Activity of Four Nano-Encapsulated Herbal Essential Oils Against Three Bacterial Fish Pathogens, Aquaculture Research. (2019) 50, no. 3, 871–875, 10.1111/are.13959, 2-s2.0-85060353015.

[145] Hudecová P. , Koščová J. , Hajdučková V. , Király J. , and Horňak P. , Antibacterial and Antibiofilm Activity of Essential Oils Against *Aeromonas* spp., Animals. (2024) 14, no. 22, 10.3390/ani14223202, 3202. 39595255 PMC11591162

[146] Van Doan H. , Soltani M. , and Leitão A. , et al.Streptococcosis a Re-Emerging Disease in Aquaculture: Significance and Phytotherapy, Animals. (2022) 12, no. 18, 10.3390/ani12182443, 2443. 36139303 PMC9495100

[147] Huang Z. , Liu X. , Jia S. , Zhang L. , and Luo Y. , The Effect of Essential Oils on Microbial Composition and Quality of Grass Carp (*Ctenopharyngodon iidellus*) Fillets During Chilled Storage, International Journal of Food Microbiology. (2018) 266, 52–59, 10.1016/j.ijfoodmicro.2017.11.003, 2-s2.0-85034833250. 29175764

[148] Kolygas M. N. , Kostou V. , and Pappas I. S. , et al.In Vitro Antibacterial Activity of Essential Oils From Medicinal Plants Against Major Fish Pathogens in Mediterranean Aquaculture, Frontiers in Aquaculture. (2025) 4, 10.3389/faquc.2025.1665877, 1665877.

[149] Bandeira Junior G. , Bianchini A. E. , and de Freitas Souza C. , et al.The Use of Cinnamon Essential Oils in Aquaculture: Antibacterial, Anesthetic, Growth-Promoting, and Antioxidant Effects, Fishes. (2022) 7, no. 3, 10.3390/fishes7030133, 133.

[150] Kačániová M. , Terentjeva M. , and Vukovic N. , et al.The Antioxidant and Antimicrobial Activity of Essential Oils Against *Pseudomonas* spp. Isolated From Fish, Saudi Pharmaceutical Journal. (2017) 25, no. 8, 1108–1116, 10.1016/j.jsps.2017.07.005, 2-s2.0-85024386101. 30166897 PMC6111119

[151] Lin Z. , An S. , and Zhou C. , et al.Effects of Eucalyptus Essential Oil on Growth, Immunological Indicators, Disease Resistance, Intestinal Morphology and Gut Microbiota in *Trachinotus ovatus* , Microorganisms. (2025) 13, no. 3, 10.3390/microorganisms13030537, 537. 40142432 PMC11944555

[152] Bektas S. , Ozdal M. , and Gürkök S. , Characterization of the Active Compounds of *Satureja hortensis* L. Aerial Parts Essential Oil and Its Antioxidant, Antimicrobial, and Anti-Biofilm Properties Against Fish Pathogenic Microorganisms, Journal of Essential Oil Bearing Plants. (2025) 28, no. 3, 591–603, 10.1080/0972060X.2025.2508238.

[153] de Souza Costa C. M. , da Cruz M. G. , and Lima T. B. C. , et al.Efficacy of the Essential Oils of *Mentha piperita*, *Lippia alba* and *Zingiber officinale* to Control the Acanthocephalan *Neoechinorhynchus buttnerae* in *Colossoma macropomum* , Aquaculture Reports. (2020) 18, 10.1016/j.aqrep.2020.100414, 100414.

[154] Alasgah A. A. , Ahmed G. E. , and Bayomi R. M. El , et al.Antibiofilm Potential of Cumin and Lemongrass Essential Oils Against Multidrug-Resistant *Vibrio parahaemolyticus* in Retailed Fish Samples, Food Control. (2025) 172, 10.1016/j.foodcont.2025.111162, 111162.

[155] Zhou S. , Yang Q. , and Dong J. , et al.Anthelmintic Efficacy of Palmarosa Oil and Curcuma Oil Against the Fish Ectoparasite *Gyrodactylus kobayashii* (monogenean), Animals. (2022) 12, no. 13, 10.3390/ani12131685, 1685. 35804584 PMC9265098

[156] Soares B. V. , Cardoso A. C. F. , and Campos R. R. , et al.Antiparasitic, Physiological and Histological Effects of the Essential Oil of Lippia Origanoides (Verbenaceae) in Native Freshwater Fish *Colossoma macropomum* , Aquaculture. (2017) 469, 72–78, 10.1016/j.aquaculture.2016.12.001, 2-s2.0-85003914598.

[157] Soares B. V. , Neves L. R. , and Oliveira M. S. B. , et al.Antiparasitic Activity of the Essential Oil of *Lippia alba* on Ectoparasites of *Colossoma macropomum* (tambaqui) and Its Physiological and Histopathological Effects, Aquaculture. (2016) 452, 107–114, 10.1016/j.aquaculture.2015.10.029, 2-s2.0-84948123937.

[158] Pereira J. d. A. , Dos Santos G. G. , and Costa D. S. , et al.Exploring the Antiparasitic and Antimicrobial Potential of *Schinus terebinthifolius* Raddi Essential Oil Against Fish and Shrimp Pathogens, Journal of Fish Diseases. (2024) 47, no. 11, 10.1111/jfd.14010, e14010. 39163500

[159] Pavone V. , Argote-Vega F. E. , and Butt W. , et al.Antibiofilm Power of Basil Essential Oil Against Fish-Originated Multidrug-Resistant Salmonella and Bacillus Spp.: Targeting Biofilms on Food Contact Surfaces, Foods. (2025) 14, no. 10, 10.3390/foods14101830, 1830. 40428609 PMC12110890

[160] Tariq S. , Wani S. , and Rasool W. , et al.A Comprehensive Review of the Antibacterial, Antifungal and Antiviral Potential of Essential Oils and Their Chemical Constituents against Drug-Resistant Microbial Pathogens, Microbial Pathogenesis. (2019) 134, 10.1016/j.micpath.2019.103580, 2-s2.0-85067633056, 103580. 31195112

[161] Hayatgheib N. , Fournel C. , Calvez S. , Pouliquen H. , and Moreau E. , In Vitro Antimicrobial Effect of Various Commercial Essential Oils and Their Chemical Constituents on *Aeromonas salmonicida* subsp. Salmonicida, Journal of Applied Microbiology. (2020) 129, no. 1, 137–145, 10.1111/jam.14622. 32119179

[162] Tural S. , Durmaz Y. , Urçar E. , and Turhan S. , Antibacterial Activity of Thyme (*Thymus vulgaris* L.), Laurel (*Lauris nnobilis* L.), Rosemary (*Rosmarinus officinalis* L.) and Parsley (*Petroselinum crispum* L.) Essential Oils Against Some Fish Pathogenic Bacteria, Acta Aquatica Turcica. (2019) 15, no. 4, 440–447, 10.22392/actaquatr.549380.

[163] Wei L. S. and Wee W. , Chemical Composition and Antimicrobial Activity of *Cymbopogon nardus* Citronella Essential Oil Against Systemic Bacteria of Aquatic Animals, Iranian Journal of Microbiology. (2013) 5, no. 2, 147–152. 23825733 PMC3696851

[164] Gormez O. and Diler O. , In Vitro Antifungal Activity of Essential Oils From Tymbra, Origanum, Satureja Species and Some Pure Compounds on the Fish Pathogenic Fungus, *Saprolegnia parasitica* , Aquaculture Research. (2014) 45, no. 7, 1196–1201, 10.1111/are.12060, 2-s2.0-84902156737.

[165] Hoskonen P. , Heikkinen J. , Eskelinen P. , and Pirhonen J. , Efficacy of Clove Oil and Ethanol Against *Saprolegnia* sp. and Usability as Antifungal Agents During Incubation of Rainbow Trout *Oncorhynchus mykiss* (Walbaum) Eggs, Aquaculture Research. (2015) 46, no. 3, 581–589, 10.1111/are.12200, 2-s2.0-84921549644.

[166] Tadese D. A. , Song C. , and Sun C. , et al.The Role of Currently Used Medicinal Plants in Aquaculture and Their Action Mechanisms: A Review, Reviews in Aquaculture. (2022) 14, no. 2, 816–847, 10.1111/raq.12626.

[167] Brasil E. M. , Figueredo A. B. , and Cardoso L. , et al.In Vitro and In Vivo Antiparasitic Action of Essential Oils of *Lippia* spp. in Koi Carp (*Cyprinus carpio*) Fed Supplemented Diets, Brazilian Journal of Veterinary Pathology. (2019) 12, no. 3, 88–100, 10.24070/bjvp.1983-0246.v12i3p88-100.

[168] Da Cunha J. A. , Sutili F. J. , and Oliveira A. M. , et al.The Essential Oil of *Hyptis mutabilis* in *Ichthyophthirius multifiliis* Infection and Its Effect on Hematological, Biochemical, and Immunological Parameters in Silver Catfish, *Rhamdia quelen* , The Journal of Parasitology. (2017) 103, no. 6, 778–785, 10.1645/16-174, 2-s2.0-85045830972. 28731834

[169] Kong Y.-D. , Li M. , and Xia C.-G. , et al.The Optimum Thymol Requirement in Diets of *Channa argus*: Effects on Growth, Antioxidant Capability, Immune Response and Disease Resistance, Aquaculture Nutrition. (2021) 27, no. 3, 712–722, 10.1111/anu.13217.

[170] Kuebutornye F. K. A. , Roy K. , Folorunso E. A. , and Mraz J. , Plant-Based Feed Additives in *Cyprinus carpio* Aquaculture, Reviews in Aquaculture. (2024) 16, no. 1, 309–336, 10.1111/raq.12840.

[171] Mehrim A. , Refaey M. , Abdelhamied A. , and Mekhemr I. , Impact of Caraway Essential Oil on Growth, Feed Utilization, and Flesh Quality of the Nile Tilapia, *Oreochromis niloticus* , Egyptian Journal of Aquatic Biology and Fisheries. (2024) 28, no. 11, 1489–1508.

[172] Abdel-Latif H. M. R. , Abdel-Tawwab M. , Khafaga A. F. , and Dawood M. A. O. , Dietary Oregano Essential Oil Improved the Growth Performance via Enhancing the Intestinal Morphometry and Hepato-Renal Functions of Common Carp (*Cyprinus carpio* L.) Fingerlings, Aquaculture. (2020) 526, 10.1016/j.aquaculture.2020.735432, 735432.

[173] Yigit N. O. , Metin S. , Didinen B. I. , Ozmen O. , Aslankoc R. , and Kara N. , Effect on Growth, Gonad Development, Health and Disease Resistance Against *Aeromonas hydrophila* of Origanum Minutiflorum Essential Oil in *Cyprinus carpio* , Animal Feed Science and Technology. (2024) 317, 10.1016/j.anifeedsci.2024.116086, 116086.

[174] Cheyadmi S. , Chadli H. , and Maadoudi M. E. , et al.Effect of Dietary Additives Based on Essential Oils of Lemongrass and Chamomile on the Zootechnical Performance and Physiological Stress Response of European Seabass (*Dicentrarchus labrax*) in Aquaculture, Fish Physiology and Biochemistry. (2025) 51, no. 6, 1–17, 10.1007/s10695-025-01580-1. 41186791

[175] Dinardo F. R. , Deflorio M. , Casalino E. , Crescenzo G. , and Centoducati G. , Effect of Feed Supplementation With *Origanum vulgare* L. Essential Oil on Sea Bass (*Dicentrarchus labrax*): A Preliminary Framework on Metabolic Status and Growth Performances, Aquaculture Reports. (2020) 18, 10.1016/j.aqrep.2020.100511, 100511.

[176] Gonçalves R. A. , Serradeiro R. , Machado M. , Costas B. , Hunger C. , and Dias J. , Interactive Effects of Dietary Fishmeal Level and Plant Essential Oils Supplementation on European Sea Bass, *Dicentrarchus labrax*: Growth Performance, Nutrient Utilization, and Immunological Response, Journal of the World Aquaculture Society. (2019) 50, no. 6, 1078–1092, 10.1111/jwas.12616, 2-s2.0-85065488477.

[177] Chung S. , Ribeiro K. , Melo J. F. B. , Teixeira D. V. , Vidal L. V. O. , and Copatti C. E. , Essential Oil From Ginger Influences the Growth, Haematological and Biochemical Variables and Histomorphometry of Intestine and Liver of Nile Tilapia Juveniles, Aquaculture. (2021) 534, 10.1016/j.aquaculture.2020.736325, 736325.

[178] Ahmad M. H. and Abdel-Tawwab M. , The use of Caraway Seed Meal as a Feed Additive in Fish Diets: Growth Performance, Feed Utilization, and Whole-Body Composition of Nile Tilapia, *Oreochromis niloticus* (L.) Fingerlings, Aquaculture. (2011) 314, no. 1–4, 110–114, 10.1016/j.aquaculture.2011.01.030, 2-s2.0-79952739034.

[179] de Souza R. C. , Baldisserotto B. , Melo J. F. B. , da Costa M. M. , de Souza E. M. , and Copatti C. E. , Dietary *Aloysia triphylla* Essential Oil on Growth Performance and Biochemical and Haematological Variables in Nile Tilapia, Aquaculture. (2020) 519, 10.1016/j.aquaculture.2019.734913, 734913.

[180] Heluy G. M. , Ramos L. R. V. , and Pedrosa V. F. , et al.Oregano (*Origanum vulgare*) Essential Oil as an Additive in Diets for Nile Tilapia (*Oreochromis niloticus*) Fingerlings Reared in Salinized Water, Aquaculture Research. (2020) 51, no. 8, 3237–3243, 10.1111/are.14658.

[181] Khalafalaa M. , Shehab S. M. , and Aboraya M. H. , et al.Herbal Essential Oils Improve Growth, Antioxidant Response, and Gene Expression in Nile Tilapia Fingerlings, Frontiers in Veterinary Science. (2025) 12, 10.3389/fvets.2025.1620632, 1620632. 41001068 PMC12458646

[182] Magouz F. I. , Amer A. A. , Faisal A. , Sewilam H. , Aboelenin S. M. , and Dawood M. A. O. , The Effects of Dietary Oregano Essential Oil on the Growth Performance, Intestinal Health, Immune, and Antioxidative Responses of Nile Tilapia Under Acute Heat Stress, Aquaculture. (2022) 548, 10.1016/j.aquaculture.2021.737632, 737632.

[183] Mansour A. T. , Arisha A. H. , and Abdelaziz R. , et al.Effects of Extended Dietary Supplementation With *Santalum album* Essential Oil on Hemato-Biochemical Changes, Innate Immune Response, Antioxidant Status, and Expression of Related Gene in Nile Tilapia (*Oreochromis niloticus*), Fish Physiology and Biochemistry. (2024) 50, no. 3, 955–971, 10.1007/s10695-024-01309-6. 38300372

[184] Mohamed R. A. , Yousef Y. M. , El-Tras W. F. , and Khalafallaa M. M. , Dietary Essential Oil Extract From Sweet Orange (*Citrus sinensis*) and Bitter Lemon (*Citrus limon*) Peels Improved Nile Tilapia Performance and Health Status, Aquaculture Research. (2021) 52, no. 4, 1463–1479, 10.1111/are.15000.

[185] Shourbela R. M. , El-Hawarry W. N. , Elfadadny M. R. , and Dawood M. A. O. , Oregano Essential Oil Enhanced the Growth Performance, Immunity, and Antioxidative Status of Nile Tilapia (*Oreochromis niloticus*) Reared Under Intensive Systems, Aquaculture. (2021) 542, 10.1016/j.aquaculture.2021.736868, 736868.

[186] Zargar A. , Rahimi-Afzal Z. , and Soltani E. , et al.Growth Performance, Immune Response and Disease Resistance of Rainbow Trout (*Oncorhynchus mykiss*) Fed *Thymus vulgaris* Essential Oils, Aquaculture Research. (2019) 50, no. 11, 3097–3106, 10.1111/are.14243, 2-s2.0-85070588348.

[187] Ghafarifarsani H. , Hoseinifar S. H. , Aftabgard M. , and Van Doan H. , The Improving Role of Savory (*Satureja hortensis*) Essential Oil for Caspian Roach (*Rutilus caspicus*) Fry: Growth, Haematological, Immunological, and Antioxidant Parameters and Resistance to Salinity Stress, Aquaculture. (2022) 548, 10.1016/j.aquaculture.2021.737653, 737653.

[188] Wang Y. , Liu Z. , Wang Z. , Xia J. , and Fu G. , Dietary *Litsea cubeba* Essential Oil Enhances Growth, Immunity, and Intestinal Health in Channel Catfish, Fish & Shellfish Immunology. (2025) 165, 10.1016/j.fsi.2025.110487, 110487. 40516798

[189] Copatti C. E. , Felix e Silva A. , Lorenzo V. P. , and Melo J. F. B. , Addition of Essential Oil From Lippia Sidoides to the Diet of Tambaqui: An Analysis of Growth, Metabolic and Blood Parameters, and Intestinal Enzymes, Aquaculture. (2022) 560, 10.1016/j.aquaculture.2022.738482, 738482.

[190] Carneiro C. L. d. S. , de Assis C. E. , and Modesto A. L. S. , et al.Oregano Essential Oil (*Origanum vulgare*) Dietary Supplementation Improved Growth Performance, Body Protein Retention and Muscle Hyperplasia of the Neotropical Catfish *Lophiosilurus alexandri* , Aquaculture Nutrition. (2021) 27, no. 4, 1221–1231, 10.1111/anu.13263.

[191] Guo X. , Zhu Z. , and Wang H. , et al.Effects of Dietary Oregano Essential Oil on Digestive Tissue Structure and Function, Antioxidant and Immune Responses and Gut Microbiota of Red Swamp Crayfish (*Procambarus clarkii*), Comparative Biochemistry and Physiology Part B: Biochemistry and Molecular Biology. (2025) 279, 10.1016/j.cbpb.2025.111100, 111100. 40268133

[192] da Silva M. I. , Pestana M. C. A. , and Marchão R. S. , et al.Dietary Supplementation With *Melaleuca alternifolia* Essential Oil Improves Survival and Resistance to Saprolegnia in Tambatinga, Semina: Ciências Agrárias. (2025) 46, no. 2, 401–416, 10.5433/1679-0359.2025v46n2p401.

[193] Cardoso L. , Owatari M. S. , and Chaves F. C. M. , et al.Dietary Supplementation With Lippia Sidoides Essential Oil Improves Organ Integrity but the Specific Activity of Antioxidant Enzymes is Dose-Dependent in *Danio rerio* , Journal of Animal Physiology and Animal Nutrition. (2024) 108, no. 2, 374–382, 10.1111/jpn.13899. 37899705

[194] Korni F. M. M. , Mohammed A. N. , and Moawad U. K. , Using Some Natural Essential Oils and Their Nano-Emulsions for Ammonia Management, Anti-Stress and Prevention of Streptococcosis in Nile Tilapia, *Oreochromis niloticus* , Aquaculture International. (2023) 31, no. 4, 2179–2198, 10.1007/s10499-023-01076-w.

[195] d. S. Sosa B. , Moro E. B. , and Gomes R. L. M. , et al.Essential Oils in Diets for Nile Tilapia Juveniles: Productive Performance and Plasmatic Biochemistry, Aquaculture Research. (2020) 51, no. 7, 2758–2765, 10.1111/are.14614.

[196] Costa T. S. , Silva R. C. d. , and Pretto A. , et al.Effect of Lippia Grata Essential Oil as a Feed Additive on the Performance of Tambatinga Juveniles, Acta Amazonica. (2022) 52, no. 2, 122–130, 10.1590/1809-4392202102442.

[197] Huyben D. , Chiasson M. , Lumsden J. S. , Pham P. H. , and Chowdhury M. A. K. , Dietary Microencapsulated Blend of Organic Acids and Plant Essential Oils Affects Intestinal Morphology and Microbiome of Rainbow Trout (*Oncorhynchus mykiss*), Microorganisms. (2021) 9, no. 10, 10.3390/microorganisms9102063, 2063. 34683384 PMC8537560

[198] Raslan W. S. , Shehab A. , and Matter A. F. , et al.Impact of Essential Oil and Probiotics Supplementation on Growth Performance, Serum Biomarkers, Antioxidants Status, Bioenergetics and Histomorphometry of Intestine of Nile Tilapia Fingerlings Challenged with *Aeromonas veronii* , BMC Veterinary Research. (2025) 21, no. 1, 10.1186/s12917-024-04433-w, 6. 39773641 PMC11706111

[199] Öz M. , Effects of Boric Acid on Oxidative Stress Parameters, Growth Performance and Blood Parameters of Rainbow Trout (*Oncorhynchus mykiss*), Biological Trace Element Research. (2025) 203, no. 3, 1647–1655, 10.1007/s12011-024-04276-4. 38913295 PMC11872762

[200] Çelik M. , Dikel S. , and Öz M. , Investigation of the Effect of Water and Feed Sourced Boron on the Growth Performance and Blood Parameters of Nile Tilapia, *Oreochromis niloticus* , Journal of the World Aquaculture Society. (2024) 55, no. 6, 10.1111/jwas.13104, e13104.

[201] Wei L. S. , Goh K. W. , Abdul Hamid N. K. , Abdul Kari Z. , Wee W. , and Van Doan H. , A Mini-Review on Co-Supplementation of Probiotics and Medicinal Herbs: Application in Aquaculture, Frontiers in Veterinary Science. (2022) 9, 10.3389/fvets.2022.869564, 869564. 36406063 PMC9666728

[202] Cao K. , Wang Y. , and Li M. , et al.Supplementation of a Multienzyme Complex, an Organic Acid-Essential Oil Complex, and Prebiotic Alone or in Combination Affects Growth, Nutrient Utilization, and Immune Function of Rainbow Trout (*Oncorhynchus mykiss*), Aquaculture Nutrition. (2022) 2022, no. 1, 10.1155/2022/1068537, 1068537.

[203] Mendes J. , Martins H. , and Otoni C. , et al.Chemical Composition and Antibacterial Activity of *Eugenia brejoensis* Essential Oil Nanoemulsions Against *Pseudomonas fluorescens* , LWT. (2018) 93, 659–664, 10.1016/j.lwt.2018.04.015, 2-s2.0-85045402602.

[204] Singh I. R. and Pulikkal A. K. , Preparation, Stability and Biological Activity of Essential Oil-Based Nano Emulsions: A Comprehensive Review, OpenNano. (2022) 8, 10.1016/j.onano.2022.100066, 100066.

[205] Li K. , Zhang M. , Bhandari B. , Xu J. , and Yang C. , Improving Storage Quality of Refrigerated Steamed Buns by Mung Bean Starch Composite Coating Enriched With Nano-Emulsified Essential Oils, Journal of Food Process Engineering. (2020) 43, no. 9, 10.1111/jfpe.13475, e13475.

[206] Shi Y. , Zhang M. , Chen K. , and Wang M. , Nano-Emulsion Prepared by High Pressure Homogenization Method as a Good Carrier for Sichuan Pepper Essential Oil: Preparation, Stability, and Bioactivity, LWT. (2022) 154, 10.1016/j.lwt.2021.112779, 112779.

[207] Valipour A. , Heidari B. , and Esmaeili Gouvarchin Ghaleh H. , et al.Enhancment of Zebrafish (*Danio rerio*) Immune and Antioxidant Systems Using Medicinal Plant Extracts Encapsulated in Alginate-Chitosan Nanocapsules With Slow Sustained Release, Biologia Futura. (2024) 75, no. 4, 437–451, 10.1007/s42977-024-00244-0. 39278890

[208] Turek C. and Stintzing F. C. , Stability of Essential Oils: A Review, Comprehensive Reviews in Food Science and Food Safety. (2013) 12, no. 1, 40–53, 10.1111/1541-4337.12006, 2-s2.0-84871985126.

[209] Pavoni L. , Benelli G. , Maggi F. , and Bonacucina G. , Green Nanoemulsion Interventions for Biopesticide Formulations, Nano-Biopesticides Today and Future Perspectives, 2019, Elsevier, 133–160.

[210] Perlatti B. , de Souza Bergo P. L. , Fernandes J. B. , and Forim M. R. , Polymeric Nanoparticle-Based Insecticides: A Controlled Release Purpose for Agrochemicals, Insecticides-Development of Safer and More Effective Technologies: IntechOpen, 2013.

[211] Cunha J. A. , Heinzmann B. M. , and Baldisserotto B. , The Effects of Essential Oils and Their Major Compounds on Fish Bacterial Pathogens – A Review, Journal of Applied Microbiology. (2018) 125, no. 2, 328–344, 10.1111/jam.13911, 2-s2.0-85047788246. 29742307

[212] Caputo A. , Bondad-Reantaso M. G. , and Karunasagar I. , et al.Antimicrobial Resistance in Aquaculture: A Global Analysis of Literature and National Action Plans, Reviews in Aquaculture. (2023) 15, no. 2, 568–578, 10.1111/raq.12741.

[213] Magara G. , Prearo M. , and Vercelli C. , et al.Modulation of Antioxidant Defense in Farmed Rainbow Trout (*Oncorhynchus mykiss*) Fed With a Diet Supplemented by the Waste Derived From the Supercritical Fluid Extraction of Basil (*Ocimum basilicum*), Antioxidants. (2022) 11, no. 2, 10.3390/antiox11020415, 415. 35204297 PMC8869336

[214] Wickramanayake M. , Kumarage P. , Majeed S. , and Heo G. , An Overview of the Antimicrobial Activity of Some Essential Oils Against Fish Pathogenic Bacteria, Veterinary Integrative Sciences. (2022) 21, no. 1, 99–119, 10.12982/VIS.2023.009.

[215] Espinoza J. , Urzúa A. , and Sanhueza L. , et al.Essential Oil, Extracts, and Sesquiterpenes Obtained From the Heartwood of *Pilgerodendron uviferum* Act as Potential Inhibitors of the *Staphylococcus aureus* NorA Multidrug Efflux Pump, Frontiers in Microbiology. (2019) 10, 10.3389/fmicb.2019.00337, 2-s2.0-85065903882, 337. 30863385 PMC6400098

[216] Milijasevic M. , Veskovic-Moracanin S. , Babic Milijasevic J. , Petrovic J. , and Nastasijevic I. , Antimicrobial Resistance in Aquaculture: Risk Mitigation Within the One Health Context, Foods. (2024) 13, no. 15, 10.3390/foods13152448, 2448. 39123639 PMC11311770

[217] Öz M. , Üstüner E. , Çifci S. , Budak F. , İleri E. , and Dikel S. , Artificial Intelligence for Fish Disease Diagnosis and Management: Innovations, Challenges, and One Health Implications, Aquaculture International. (2025) 33, no. 7, 1–36, 10.1007/s10499-025-02352-7.

[218] Jaber H. , Fikraoui N. , Zaazoui N. , Bourkhiss B. , and Ouhssine M. , Chemical Composition, Organoleptic, Physicochemical, and Antibacterial Properties of Three Plants of the Lamiaceae Family on *Escherichia Coli* Strains, E3S Web of Conferences. (2024) 527, 01015.

[219] Mancuso M. , Catalfamo M. , and Laganà P. , et al.Screening of Antimicrobial Activity of Citrus Essential Oils Against Pathogenic Bacteria and Candida Strains, Flavour and Fragrance Journal. (2019) 34, no. 3, 187–200, 10.1002/ffj.3491, 2-s2.0-85061780482.

[220] Mohd Israfi N. A. , Mohd Ali M. I. A. , and Manickam S. , et al.Essential Oils and Plant Extracts for Tropical Fruits Protection: From Farm to Table, Frontiers in Plant Science. (2022) 13, 10.3389/fpls.2022.999270, 999270. 36247633 PMC9559231

[221] D’Aquila P. , Paparazzo E. , and Crudo M. , et al.Antibacterial Activity and Epigenetic Remodeling of Essential Oils From Calabrian Aromatic Plants, Nutrients. (2022) 14, no. 2, 10.3390/nu14020391, 391. 35057572 PMC8780331

[222] Rezzoug M. , Bakchiche B. , and Gherib A. , et al.Chemical Composition and Bioactivity of Essential Oils and Ethanolic Extracts of *Ocimum basilicum* L. and Thymus Algeriensis Boiss. & Reut. From the Algerian Saharan Atlas, BMC Complementary and Alternative Medicine. (2019) 19, no. 1, 10.1186/s12906-019-2556-y, 2-s2.0-85068503845, 146. 31227024 PMC6588939

[223] Serradell A. , Montero D. , and Terova G. , et al.Functional Additives in a Selected European Sea Bass (*Dicentrarchus labrax*) Genotype: Effects on the Stress Response and Gill Antioxidant Response to Hydrogen Peroxide (H2O2) Treatment, Animals. (2023) 13, no. 14, 10.3390/ani13142265, 2265. 37508043 PMC10376812

[224] Sun Y. , McDonald T. , and Baur A. , et al.Fish Oil Supplementation Modifies the Genetic Potential for Blood Lipids, medRxiv. (2023) 10.1101/2023.09.22.23295987, 2023.09.22.23295987.

[225] Kalyana Babu B. , Mary Rani K. L. , and Sahu S. , et al.Development and Validation of Whole Genome-Wide and Genic Microsatellite Markers in Oil Palm (*Elaeis guineensis* Jacq.): First Microsatellite Database (OpSatdb), Scientific Reports. (2019) 9, no. 1, 10.1038/s41598-018-37737-7, 2-s2.0-85061508682, 1899. 30760842 PMC6374426

[226] Osorio-Guarín J. A. , Garzón-Martínez G. A. , and Delgadillo-Duran P. , et al.Genome-Wide Association Study (GWAS) for Morphological and Yield-Related Traits in an Oil Palm Hybrid (*Elaeis oleifera* x *Elaeis guineensis*) Population, BMC Plant Biology. (2019) 19, no. 1, 10.1186/s12870-019-2153-8, 533. 31795941 PMC6889324

